# Host cell sensing and restoration of mitochondrial function and metabolism within *Helicobacter pylori* VacA intoxicated cells

**DOI:** 10.1128/mbio.02117-23

**Published:** 2023-10-10

**Authors:** Ami Y. Seeger, Faisal Zaidi, Sammy Alhayek, Rachel M. Jones, Huzaifa Zohair, Robin L. Holland, Ik-Jung Kim, Steven R. Blanke

**Affiliations:** 1 Department of Microbiology, University of Illinois, Urbana, Illinois, USA; 2 Department of Pathobiology, University of Illinois, Urbana, Illinois, USA; 3 Buck Institute for Research on Aging, Novato, California, USA; 4 Department of Biomedical and Translational Medicine, University of Illinois, Urbana, Illinois, USA; University of Oklahoma Health Sciences Center, Oklahoma City, Oklahoma, USA

**Keywords:** *Helicobacter pylori*, vacuolating cytotoxin, VacA, mitochondrial dysfunction, mitochondrial dynamics, proton motive force, transmembrane potential, dynamin-related protein 1, AMPK, mitochondrial depolarization

## Abstract

*Helicobacter pylori* vacuolating cytotoxin A (VacA) is an intracellular-acting protein exotoxin that induces mitochondrial dysfunction and energy depletion within host cells. Although exposure to VacA results in mitochondrial dysfunction, one recent study revealed that, following limited exposure to VacA, mitochondrial function and cellular ATP levels were restored in a time-dependent manner. Studies performed to address the mechanism by which host cells detect and respond to intracellular VacA identified the adenosine monophosphate (AMP)-activated protein kinase (AMPK) as a sensor of toxin-dependent alterations in cellular energy status. Activation of AMPK in response to VacA was demonstrated to orchestrate alterations in mitochondrial dynamics which resulted in restoration of mitochondrial function. Specifically, upregulation of dynamin-related protein 1 (Drp-1)-dependent mitochondrial fission resulted in reversible fragmentation of filamentous mitochondria and time-dependent reduction in mitochondrial-associated VacA, suggesting that fragmentation is important for removal of VacA from mitochondria. Cells with reduced levels of Drp-1 were more susceptible to VacA-dependent cell death, suggesting that mitochondrial dynamics is important for maintaining cell viability through the reduction in mitochondrial-associated toxin. Collectively, these studies support a model that cellular recovery and survival in response to VacA-dependent mitochondrial dysfunction is linked to host cell modulation of mitochondrial dynamics. This study provides new insights into cellular recognition and responses to intracellular-acting toxin modulation of host cell function, which could be relevant for the growing list of pathogenic microbes and viruses identified that target mitochondria as part of their virulence strategies.

**IMPORTANCE:**

Persistent human gastric infection with *Helicobacter pylori* is the single most important risk factor for development of gastric malignancy, which is one of the leading causes of cancer-related deaths worldwide. An important virulence factor for *Hp* colonization and severity of gastric disease is the protein exotoxin VacA, which is secreted by the bacterium and modulates functional properties of gastric cells. VacA acts by damaging mitochondria, which impairs host cell metabolism through impairment of energy production. Here, we demonstrate that intoxicated cells have the capacity to detect VacA-mediated damage, and orchestrate the repair of mitochondrial function, thereby restoring cellular health and vitality. This study provides new insights into cellular recognition and responses to intracellular-acting toxin modulation of host cell function, which could be relevant for the growing list of pathogenic microbes and viruses identified that target mitochondria as part of their virulence strategies.

## INTRODUCTION

Chronic infection with the human gastric pathogen *Helicobacter pylori* (*Hp*) is the single most important risk factor for development of gastric cancer (1, 2). *Hp* persistently infects approximately 50% of the global population, and approximately 1% of chronic infections progress to malignancy. However, the infection biology and virulence strategies underlying chronic *Hp* infection within the gastric environment remains poorly understood. Vacuolating cytotoxin A (VacA) is an intracellular-acting protein exotoxin secreted by most *Hp* strains (3, 4) that interact with and enter host cell within the gastric glands (5 – 10). Although our understanding of the exact role of VacA in *Hp* pathogenesis is not fully understood, animal models of infection have implicated VacA as an important determinant of *Hp* colonization (11, 12).

Internalization of VacA by gastric epithelial cells results in toxin targeting of the mitochondrial inner membrane (13 – 16), resulting in dissipation of transmembrane potential (ΔΨ_m_) (17, 18). The ensuing collapse of proton motive force reduces cellular ATP production (4, 17) , thereby disrupting metabolic homeostasis, as indicated by toxin-mediated inhibition of mTORC1 (19) and activation of autophagy (19 – 22). Because dissipation of ΔΨ_m_ can negatively impact cell vitality (23 – 26), we originally predicted that VacA-dependent loss of proton motive force might be associated with previously reported gastric tissue damage and cell death during *Hp* infection (15, 27, 28). However, recent *in vivo* mouse studies demonstrated that extended intragastric infusion of purified VacA did not result in major tissue damage or an increase in cell death within the gastric epithelium (29). In addition, recent *in vitro* cell culture studies have also demonstrated that cell viability is largely retained following exposure to VacA at toxin concentrations we have found to be sufficient to induce mitochondrial dysfunction (19, 30). Overall, the mechanism by which host cells are able to survive VacA-mediated mitochondrial damage and reduction in cellular energy remains poorly understood.

Here, we hypothesized that host cells possess a mechanism for responding to the presence of intracellular VacA by sensing the impact of toxin-mediated mitochondrial damage. Recent work supports this hypothesis through the demonstration that mitochondrial function is restored in a time-dependent manner following limited exposure to VacA, although the mechanism underlying mitochondrial restoration was not identified (31). The studies described herein demonstrate that mitochondrial function and metabolic homeostasis within VacA-intoxicated cells are restored by a mechanism associated with modulation of mitochondrial structural dynamics. Sensing of cellular energy status via adenosine monophosphate (AMP)-activated protein kinase (AMPK) was identified to be important for activation of the mitochondrial fission machinery, which in turn was demonstrated to be important for time-dependent reduction in mitochondrial-associated VacA and increased cell viability. Overall, these studies provide new insights into the fate of intracellular VacA during *Hp* infection and provide the framework for understanding how host cells potentially sense and react to members of a growing list of pathogenic microbes and viruses identified to target mitochondria as part of their virulence strategies (32, 33).

## RESULTS

### Restoration of mitochondrial function within VacA intoxicated cells

**Fig 1:**
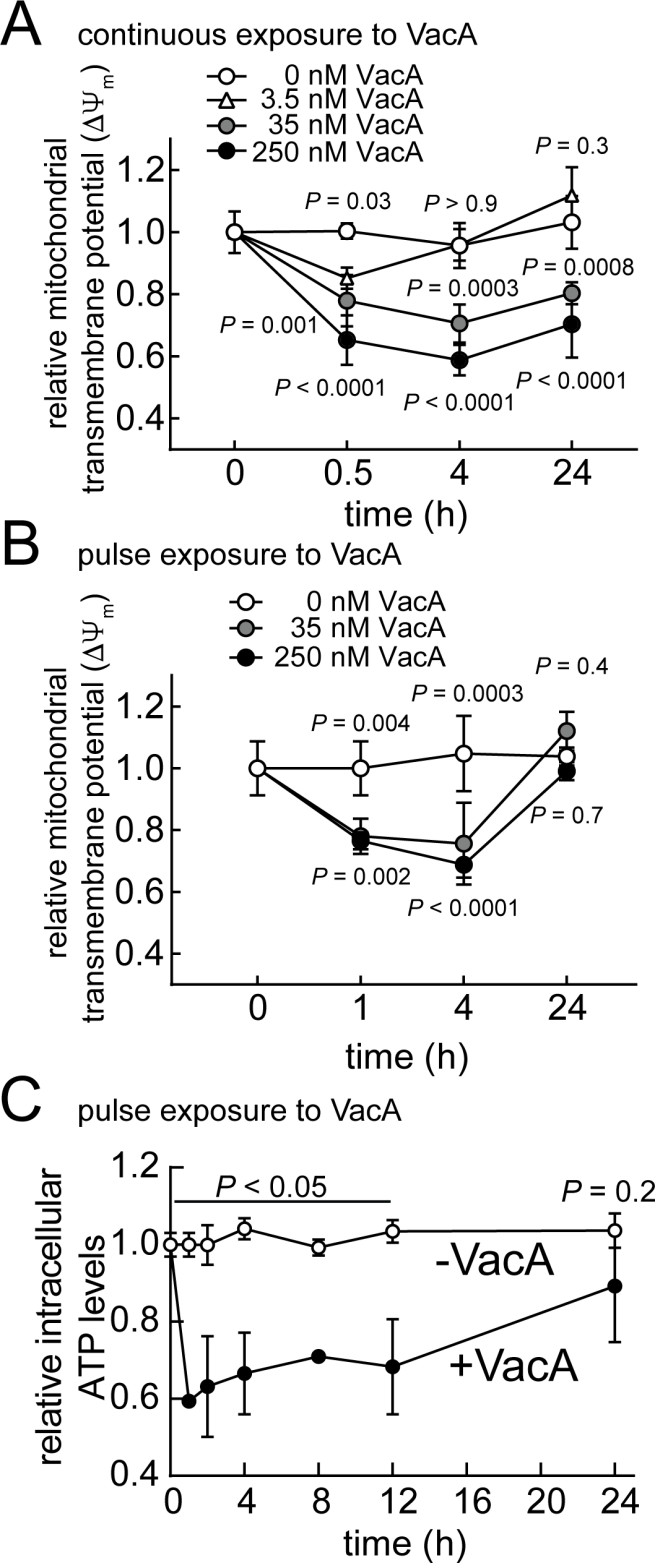
VacA-mediated mitochondrial dysfunction is restored in a time- and concentration-dependent manner. AZ-521 cells were incubated in the absence or presence of VacA (3.5, 35, 250 nM), which we have defined as “continuous exposure” of cells to VacA (**A**). Alternatively, AZ-521 cells were incubated for 10 minutes in the absence or presence of VacA [35 (**B**) or 250 nM (**B and C**)],which we have defined as “pulse exposure” of cells to VacA. After 10 minutes, cells were washed twice in PBS (pH 7.4) to eliminate any unbound extracellular VacA and further incubated in the presence of fresh cell culture medium for the duration of the experiment (**B and C**). Ten (**A**) or 30 (**B**) minutes prior to collection, cells were stained with Tetramethylrhodamine, ethyl ester (TMRE). Stained cells were evaluated for mitochondrial transmembrane potential (ΔΨ_m_) using flow cytometry. Intracellular ATP levels were determined using a luminescence-based assay according to the manufacturer’s instructions (**C**). The fluorescence or luminescence values of intoxicated cells were compared relative to unintoxicated cells. Error bars for several data points were obscured due to low variance. The data were combined from three independent experiments (± SD). Statistical significance (α = 0.05) was calculated using two-way ANOVA with Dunnett’s multiple comparisons test (**A and B**) or with Sidak’s multiple comparisons test (**C**). *P* < 0.05 indicates statistical significance.

VacA disrupts metabolic homeostasis within epithelial cells by causing mitochondrial dysfunction (15, 17, 18). However, recent work indicated that intoxicated cells are capable of limiting toxin-dependent impairment of mitochondrial-mediated metabolism (31). To begin defining the mechanism by which host cells curb mitochondrial damage generated by VacA, we monitored toxin-dependent alterations in mitochondrial transmembrane potential (ΔΨ_m_). Human duodenal-derived epithelial AZ-521 cells (34 – 36) or human gastric-derived epithelial AGS cells (30, 36, 37), both of which have been commonly used to study VacA toxin biology, were incubated continuously at 37°C and under 5% CO_2_ in the absence or presence of VacA. After 0.5, 4, or 24 hours, cells that had been stained with Tetramethylrhodamine, ethyl ester (TMRE) were collected and examined for ΔΨ_m_ using flow cytometry. At 30 minutes, significant ΔΨ_m_ dissipation was detected within AZ-521 cells that had been exposed to VacA (3.5, 35, 250 nM) (Fig. 1A). At higher toxin concentrations (35 nM and 250 nM), loss of ΔΨ_m_ did not progress in an unabated manner, but instead leveled off and was sustained throughout the duration of the experiment (up to 24 hours). However, in cells exposed to a lower concentration of VacA (3.5 nM), ΔΨ_m_ had increased back to levels measured in the control (i.e., unintoxicated) cells, suggesting that mitochondrial function had been restored. These trends were reproducible in AGS cells (Fig. S1A).

We also examined the restoration of mitochondrial function where the exposure of cells to VacA was restricted, using what we refer to as “pulse exposure” of monolayers to toxin. In these experiments, cells were incubated for 10 minutes at 37°C and under 5% CO_2_ in the absence or presence of VacA (35 or 250 nM). After 10 minutes, cells were washed to remove non-cell associated VacA, and then further incubated in the presence of fresh cell culture medium (without toxin) for the duration of the experiment. In cells that had undergone pulse exposure to VacA, significant ΔΨ_m_ dissipation was initially observed, but a full recovery in mitochondrial transmembrane potential was measured after 24 hours (Fig. 1B; Fig. S1B), suggesting that mitochondrial function had been restored in cells with limited exposure to VacA. Also consistent with previous work (17, 28, 31), exposure to VacA under restricted conditions resulted in an initial reduction in cellular ATP, as expected from toxin-mediated uncoupling of oxidative phosphorylation, followed by time-dependent recovery in cellular ATP levels (Fig. 1C). Overall, these data are consistent with earlier studies (31) reporting that mitochondrial dysfunction resulting from continuous exposure to low concentrations of VacA is reversible.

Historically, *in vitro* assays of cellular changes resulting from exposure to VacA have been performed in the presence of weak bases (e.g*.,* NH_4_Cl) in part because several cellular phenotypes associated with the toxin, including cellular vacuolation and cell death, are augmented in the presence of weak bases (30). However, recent *in vivo* and *in vitro* studies demonstrated that extended exposure to VacA in the absence of weak bases results in alteration of host cellular metabolism, while at the same time causing little to no physiological damage to the gastric mucosa or cell death in animals (19, 29). Here, we found that the presence of NH_4_Cl (5 mM) did not detectably alter VacA-dependent ΔΨ_m_ dissipation and subsequent recovery of mitochondrial function (Fig. S2A).

### Mitophagy is not associated with VacA-dependent mitochondrial dysfunction

One mechanism by which cells respond to damaged mitochondria is by induction of mitochondrial-specific autophagy, called mitophagy. To evaluate whether mitophagy is induced in cells under the same conditions in which we observed VacA-dependent mitochondrial damage and recovery, we monitored mitochondrial mass, which decreased in response to mitophagy activation (38 – 40). Immunoblot analyses revealed that cellular levels of TIM23 and VDAC, which are commonly used as inner and outer mitochondrial membrane markers, respectively, were not significantly different in cells that had been incubated in the absence or presence of VacA (Fig. S3). In contrast, significant losses in cellular levels of both TIM23 and VDAC were detected in response to the known chemical activators of mitophagy, carbonyl cyanide m-chlorophenylhydrazone (CCCP), or a combination of Antimycin A and Oligomycin A (Fig. S3). These results suggest that restoration of mitochondrial function in cells exposed to VacA is not dependent upon activation of the mitophagic response.

### Exposure to VacA activates AMPK, a central sensor of cellular energy status

**Fig 2:**
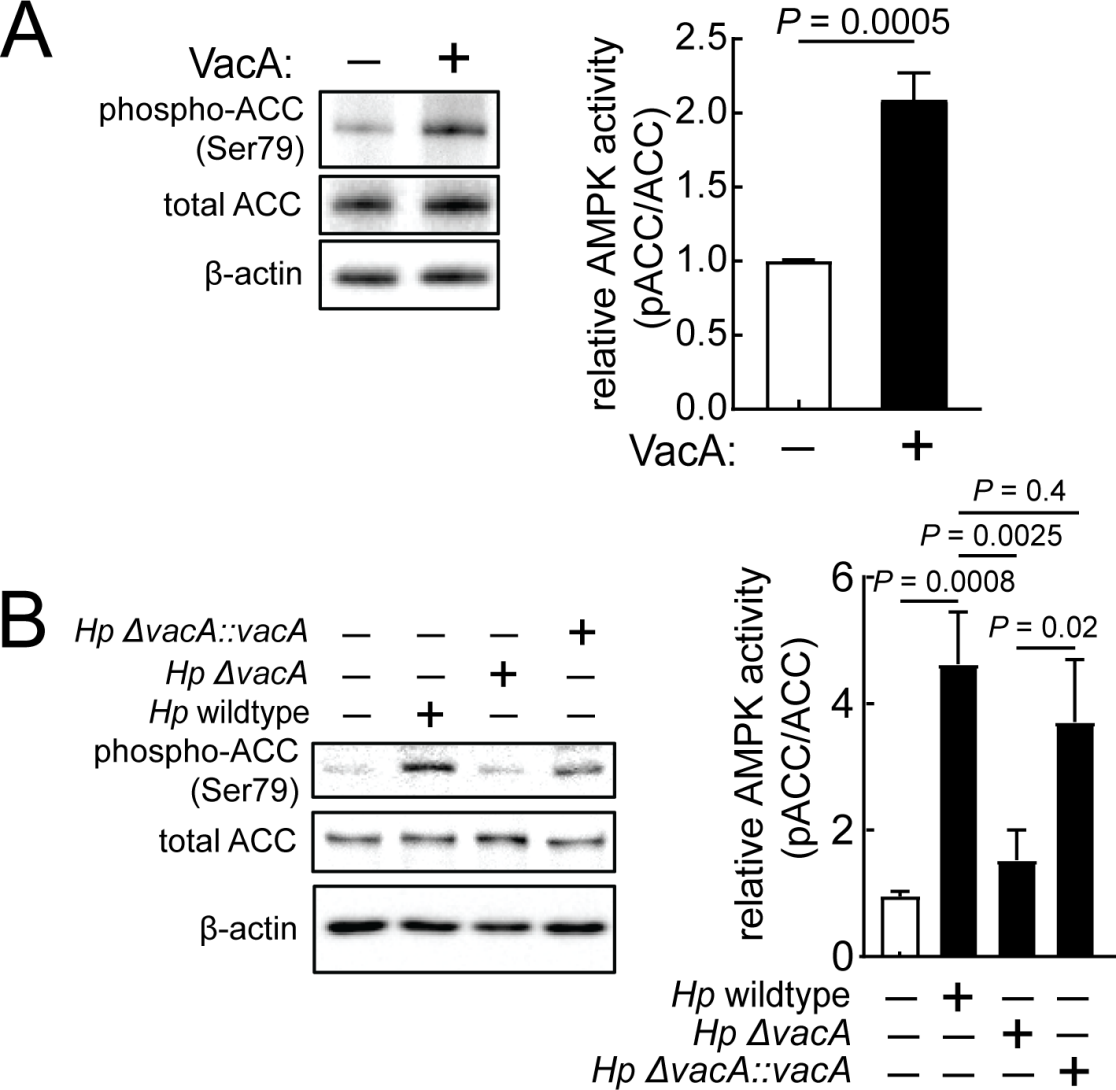
VacA-mediated cellular energy reduction activates AMPK. AZ-521 cells were incubated in the absence or presence of VacA (250 nM) (**A**). Alternatively, AZ-521 cells were incubated in the absence or presence of *Hp* 60190 (wild type), *Hp* (*ΔvacA*), or *Hp* (*ΔvacA::vacA*) at MOI 100 (**B**). After 2 hours (**A**) or 1 hour (**B**), cell lysates were collected and evaluated using immunoblot analysis to determine the relative levels of phospho-ACC (S79). Immunoblots representative of those collected from three independent experiments are shown. Quantification of the band intensities were evaluated by densitometry. The data were combined from three independent experiments (represented as ±SD). Statistical significance (α = 0.05) was calculated using unpaired *t*-test (**A**) or one-way ANOVA with Tukey’s multiple comparisons test (**B**). *P* < 0.05 indicates statistical significance.

The reversibility of VacA-dependent mitochondrial dysfunction suggests that intoxicated cells must possess a mechanism which recognizes and responds to the consequences of ΔΨ_m_ dissipation. Within mammalian cells, adenosine monophosphate-activated protein kinase functions as a central sensor of cellular energy status (41 – 43). Assessment of possible AMPK involvement in response to continuous exposure to VacA revealed a significant toxin-dependent increase in phosphorylated acetyl-CoA carboxylase (ACC) [p-ACC (S79)], which is a marker for AMPK activation (Fig. 2A; Fig. S4A and B). Notably, AMPK activation was observed when AZ-521 cells were exposed to VacA at concentrations as low as 3.5 nM (Fig. S4C), which is sufficient to induce the dissipation of ΔΨ_m_ (Fig. 1A).

AMPK was also activated in AZ-521 cells infected with *Hp* 60190, which secrete active VacA, but was not activated during infection with a mutant strain of *Hp* 60190 lacking the gene encoding functional VacA [*Hp* 60190 (*ΔvacA*)] (Fig. 2B). Infection with the complemented strain, *Hp* 60190 (*ΔvacA::vacA*), resulted in AMPK activation to similar levels as those induced during infection with the wild-type strain. These results confirmed that AMPK is activated during *in vitro Hp* infection of cells in a manner dependent upon VacA.

To further establish the importance of VacA for activation of AMPK during *Hp* infection, we examined the effects of perturbing toxin structure-function relationships previously demonstrated to be critical for toxin cellular activity. VacA monomers assemble into higher-ordered six- or seven-membered rings (44 – 48) that are essential for the formation of ion-conducting membrane channels (15, 18, 49 – 51). In our studies, AMPK activation was attenuated in cells infected with modified strains of *Hp* 60190 expressing mutant forms of VacA that are deficient in either membrane channel-forming activity [e.g*., Hp vacA* (P9A) and *Hp vacA* (G14A)] (Fig. S4D), or the capacity to assemble into higher-ordered structures [e.g.*, Hp vacA* (Δ49–57)*, Hp vacA* (Δ346–347)*,* and *Hp vacA* (Δ49–57Δ346–347)] (Fig. S4E), which are both required for VacA cellular intoxication (45, 52 – 56). These results support a model that *Hp*-dependent AMPK activation results from the dissipation of ΔΨ_m_ caused by the membrane channel-forming activity of VacA.

### VacA-dependent AMPK activation precedes restoration of mitochondrial function

Studies to assess the time-dependent association between VacA-mediated mitochondrial dysfunction and AMPK activation revealed a significant increase in cellular levels of p-ACC (S79) within cells exposed to VacA at 0.5 hour (Fig. S4F), a timepoint at which significant dissipation of mitochondrial transmembrane potential (ΔΨ_m_) is robustly measured in cells that had either continuous or limited exposure to toxin. The cellular levels of p-ACC (S79) remained significantly elevated through 8 hours in cells exposed continuously to VacA, indicating that prolonged AMPK activation is associated with sustained mitochondrial dysfunction. In cells with limited (i.e*.,* pulse) exposure to toxin, cellular p-ACC (S79) levels were significantly increased at 15 minutes and reached maximum levels after 60 minutes (Fig. S4G), similar to cells that had been continuously exposed to VacA. In contrast, activated AMPK was no longer detected in monolayers with limited exposure to toxin at 4 hours, a timepoint before recovery of ΔΨ_m_ was observed (Fig. 1B), indicating that AMPK activation precedes restoration of mitochondrial function.

### AMPK activation is important for restoration of mitochondrial function in VacA-intoxicated cells

**Fig 3:**
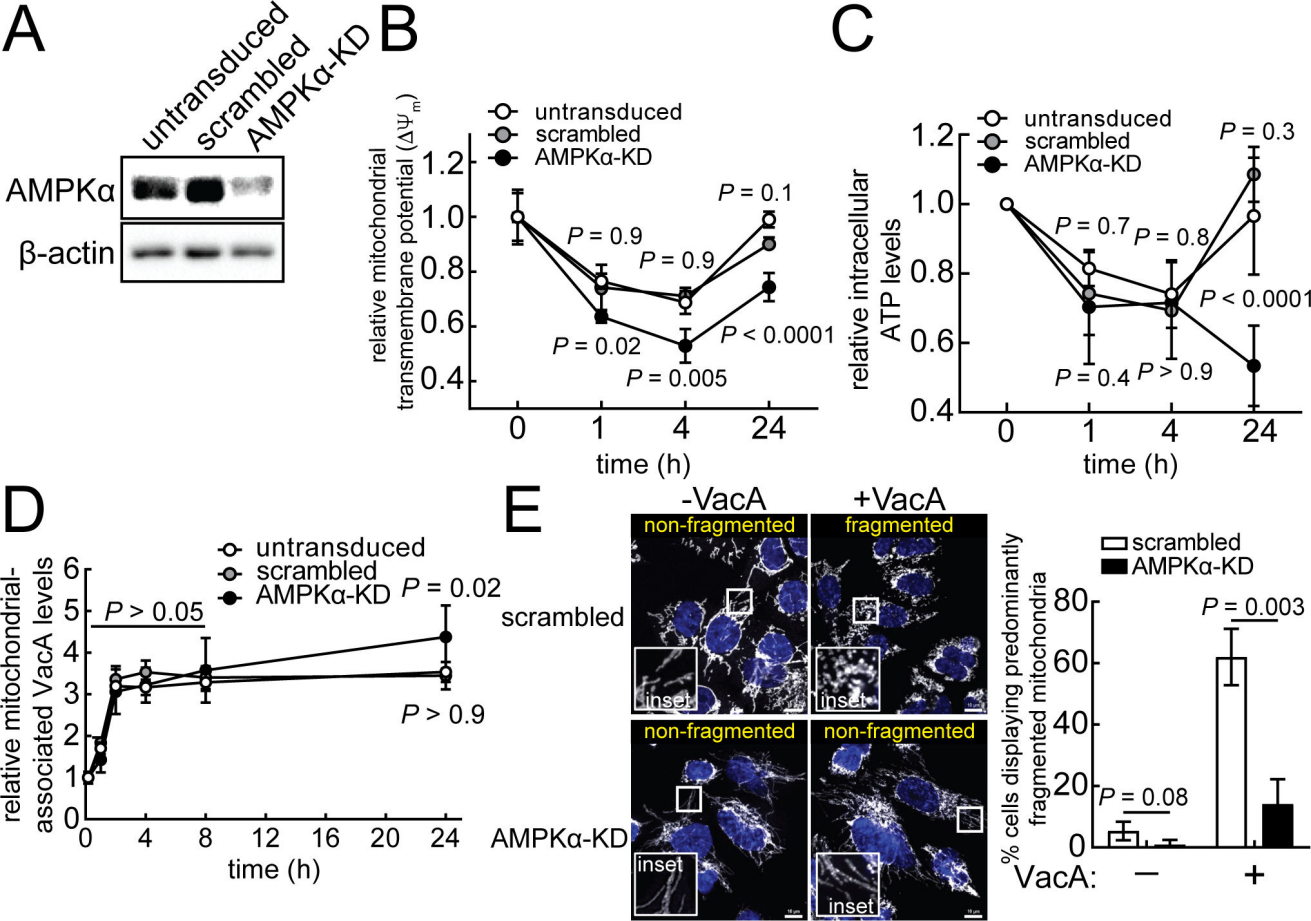
AMPK is important for mitochondrial functional restoration. AMPKα-knockdown (AMPKα-KD) in AZ-521 cells were generated and confirmed using immunoblot analysis of cell lysates (**A**). Untransduced, scrambled, or AMPKα-KD cells were incubated in the absence or presence of VacA (250 nM) using pulse (**B and C**) or continuous (**D and E**) toxin exposure. Cells were stained with TMRE for evaluating mitochondrial transmembrane potential (ΔΨ_m_) (**B**). Intracellular ATP levels were determined using a luminescence-based assay (**C**). The data from intoxicated cells were compared relative to unintoxicated cells (measured at 0 hour). Statistical significance was determined by comparing data from transduced cells and untransduced cells at the indicated timepoints. Because the data in Fig. 3B and Fig. 4B, as well as Fig. 3C and Fig. 4C, were collected simultaneously, the controls (i.e., untransduced cells and cells transduced with scrambled shRNA) are identical in both figures. For determining relative levels of mitochondrial-associated VacA (**D**), mitochondrial fractions were assessed for VacA and TIM23. Immunoblots representative of those collected from three independent experiments are found in Fig. S7. The data represented in (**D**) were rendered relative to the 10-minute timepoint. For evaluating mitochondrial structure (**E**), cells were fixed after 1 hour and evaluated by fluorescence microscopy analysis using antibodies specific for TOM20. Fluorescence images (White = TOM20; Blue = DAPI) shown are representative of three independent experiments. Scale bars indicate 10 µm. The data were combined from the three independent experiments (± SD). Statistical significance (α = 0.05) was calculated using two-way ANOVA with Tukey’s multiple comparisons test (B to D) or unpaired *t*-test (**E**). *P* < 0.05 indicates statistical significance.

To assess the importance of VacA-dependent AMPK activation for mitochondrial functional recovery, we examined the time-dependent restoration of mitochondrial transmembrane potential and cellular energy within VacA-intoxicated AZ-521 (Fig. 3A) or AGS cells (Fig. S5A) with shRNA-mediated reduced levels of AMPKα, the catalytic subunit of AMPK, which we will hereafter refer to as AMPKα-knockdown (AMPKα-KD) cells. One hour after pulse exposure to VacA, ΔΨ_m_ (Fig. 3B; Fig. S5C) and cellular ATP levels (Fig. 3C) were similar in AMPKα-KD and control cells (i.e*.,* cells transduced with non-specific scrambled shRNA or untransduced cells), indicating that AMPK activity is not essential for initial toxin-mediated mitochondrial dysfunction. In contrast, restoration of mitochondrial function was significantly lower in AMPKα-KD cells than control cells 24 hours after pulse exposure to VacA (Fig. 3B and C; Fig. S5C), indicating that restoration of mitochondrial function was impaired in cells with lower AMPK activity. Notably, whereas partial restoration of ΔΨ_m_ at 24 hours was observed in AMPKα-KD cells (Fig. 3B), cellular ATP levels were lower at 24 hours than 4 hours (Fig. 3C). Although the reasons underlying the apparent incongruence between ΔΨ_m_ and ATP restoration in AMPKα-KD cells is not entirely clear, one potential explanation is that the absence of the catalytic subunit (i.e*.,* AMPKα) impairs normal AMPK-mediated regulation of energy-consuming and energy-generating activities needed to reestablish energy homeostasis, as previously observed in unrelated work (57). Regardless, these results support a role for AMPK as an important determinant in restoration of mitochondrial function in cells intoxicated with VacA.

**Fig 4:**
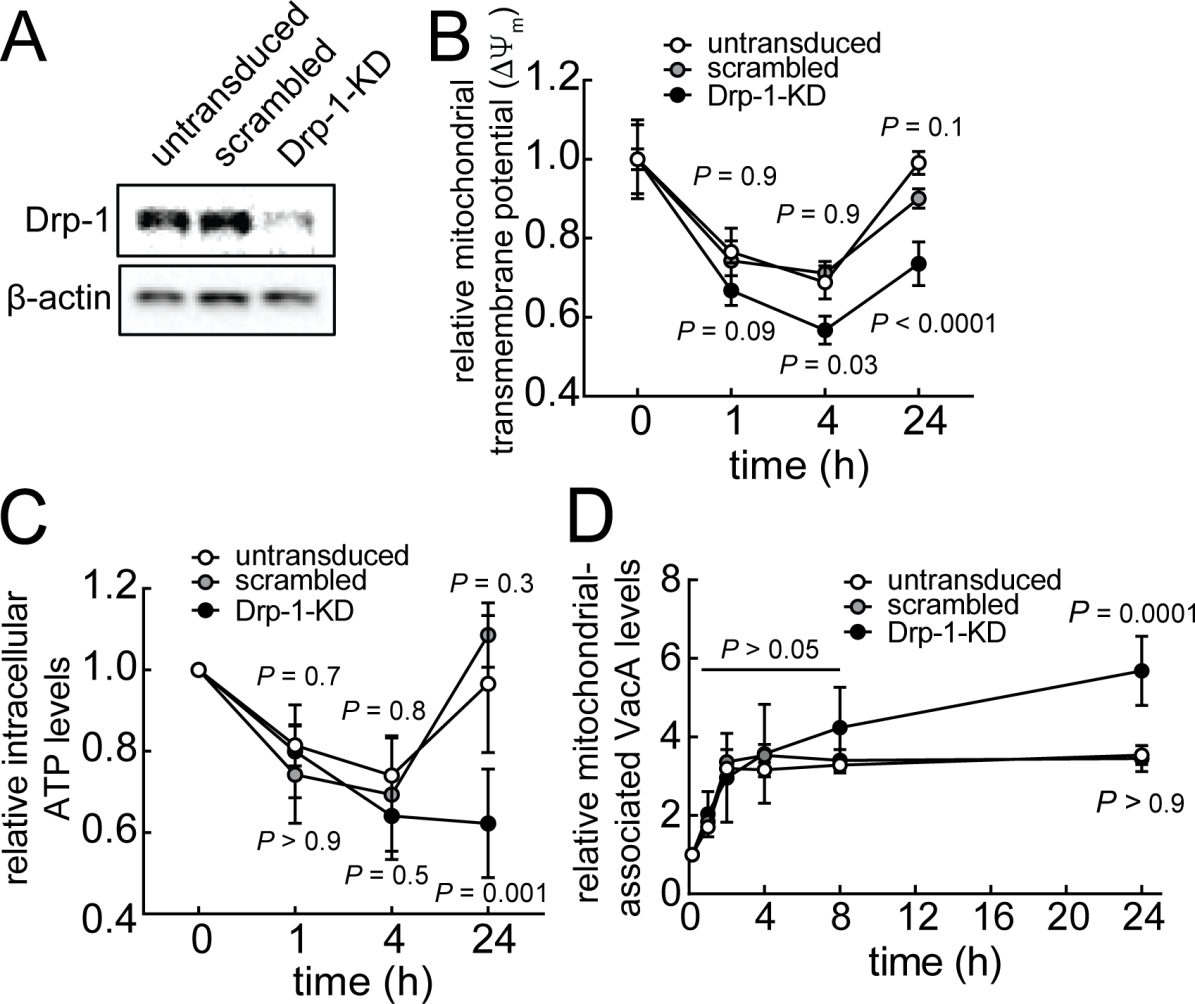
VacA-mediated mitochondrial fragmentation is important for mitochondrial functional recovery and limiting toxin accumulation in mitochondria. Drp-1-knockdown (Drp-1-KD) in AZ-521 cells were generated and confirmed using immunoblot analysis of cell lysates (**A**). Untransduced, scrambled, or Drp-1-KD cells were incubated in the absence or presence of VacA (250 nM) using pulse (**B and C**) or continuous (**D and E**) toxin exposure. Cells were stained with TMRE for evaluating mitochondrial transmembrane potential (ΔΨ_m_) (**B**).Intracellular ATP levels were determined using a luminescence-based assay (**C**). The data from intoxicated cells were compared relative to unintoxicated cells (measured at 0 hour). Statistical significance was determined by comparing data from transduced cells and untransduced cells at the indicated timepoints. Because the data in Fig. 3B and Fig. 4B, as well as Fig. 3C and Fig. 4C, were collected simultaneously, the controls (i.e., untransduced cells and cells transduced with scrambled shRNA) are identical in both figures. For determining relative levels of mitochondrial-associated VacA (**D**), mitochondrial fractions were assessed for VacA and TIM23. Immunoblots representative of those collected from three independent experiments are found in Fig. S7. The data represented in (**D**) were rendered relative to the 10-minute timepoint. The data were combined from three independent experiments (± SD). Statistical significance (α = 0.05) was determined by comparing transduced cells to untransduced cells and was calculated using two-way ANOVA with Tukey’s multiple comparisons test (B to D). *P* < 0.05 indicates statistical significance.

### Reduction of mitochondrial-associated VacA is associated with restoration of mitochondrial function

**Fig 5:**
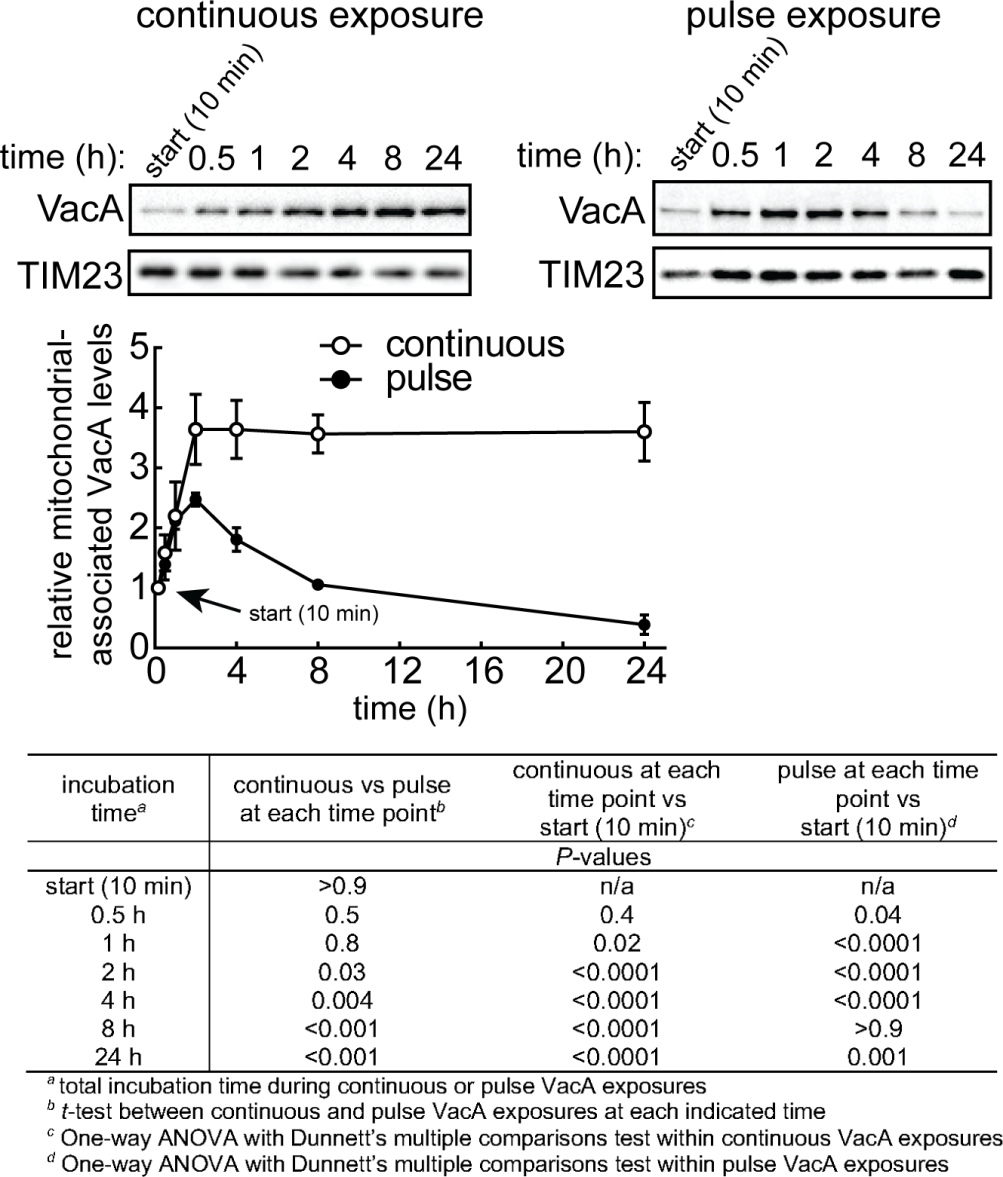
Time-dependent VacA association with mitochondria. AZ-521 cells were incubated in presence of VacA (250 nM) for continuous or pulse toxin exposures. Mitochondrial fractions were assessed by immunoblot analysis using antibodies specific for VacA and TIM23 (loading control) to determine the relative levels of mitochondrial-associated VacA. Quantification of the band intensities was evaluated by densitometry, and the values were compared relative to the 10-minute timepoint. VacA was not detected in mitochondrial fractions from cells incubated in the absence of VacA. The data were combined from three independent experiments (± SD). Statistical significance (α = 0.05) was determined by comparing continuous vs pulse at each timepoint or by comparing each timepoint vs start (10 minutes) and was calculated by *t*-test or one-way ANOVA Dunnett’s multiple comparisons test, respectively, as indicated in the table. *P* < 0.05 indicates statistical significance.

To better understand the mechanism by which mitochondrial restoration is restored in VacA intoxicated cells, we examined the time-dependent association of intracellular toxin with mitochondria (14, 16, 18, 58, 59) using immunoblot analysis of homogenized mitochondria isolated from cell lysates (Fig. S6A). For cells continuously exposed to toxin, mitochondrial-associated VacA was detected after 30 minutes, and continued to increase through 2 hours, at which point, mitochondrial-associated toxin remained relatively constant through 24 hours (Fig. 5). For cells with limited (i.e*.,* pulse) exposure to VacA, mitochondrial-associated toxin levels after 1 hour were similar to that observed in monolayers continuously exposed to toxin (Fig. 5). However, after 2 hours, we observed a sharp decline in mitochondrial-associated toxin. By 8 hours, the level of mitochondrial-associated toxin was reduced and similar to levels found after the initial 10 minutes of VacA exposure. Time-dependent reduction in mitochondrial-associated VacA was also observed in the absence or presence of NH_4_Cl (Fig. S2B and S6B). In addition, non-mitochondrial-associated intracellular VacA levels also declined in a time-dependent manner (Fig. S6C). Collectively, these data suggest that restoration of mitochondrial function within VacA intoxicated cells may involve a mechanism which limits and/or reverses toxin accumulation at mitochondria.

### AMPK activation is important for limiting VacA accumulation at mitochondria

The results described above suggest that AMPK activation (Fig. 2; Fig. S4) and the capacity of the cell to limit VacA accumulation at the mitochondria (Fig. 5) may both contribute to restoration of mitochondrial function within intoxicated cells. Experiments to evaluate this possibility revealed that, while mitochondrial-associated toxin remains relatively constant from approximately 2 to 24 hours within cell continuously exposed to VacA, mitochondrial-associated toxin in AMPKα-KD cells increased between 2 and 24 hours (Fig. 3D; Fig. S7A through C). Differences in non-mitochondrial-associated VacA levels between AMPKα-KD cells and cells transduced with non-specific (i.e*.,* scrambled) shRNA were not significant, suggesting that VacA does not likely accumulate in cellular compartments other than mitochondria in the absence of AMPK (Fig. S6D). These results are consistent with a model that AMPK sensing of cellular energy status is important for limiting and/or reversing VacA accumulation at mitochondria.

### AMPK activation is associated with mitochondrial fragmentation in VacA-intoxicated cells

In addition to mitochondrial dysfunction, fragmentation of mitochondrial structure has also been reported in cells exposed continuously to VacA by a mechanism involving modulation of mitochondrial dynamics through upregulation of dynamin-related protein-1 (Drp-1)-dependent mitochondrial fission (58). Because our studies described here identified the importance of AMPK for recovery of mitochondrial function within VacA intoxicated cells (Fig. 3B and C), we next assessed the impact, if any, of AMPK on toxin modulation of mitochondrial structural dynamics. These studies revealed an approximate fivefold decrease in VacA-mediated mitochondrial fragmentation in AMPKα-KD cells relative to control cells (Fig. 3E). VacA-mediated mitochondrial fragmentation was also inhibited in cells incubated in the presence of the AMPK inhibitor, Compound C (Fig. S8). The presence of NH_4_Cl neither inhibited nor promoted fragmentation of filamentous mitochondria (Fig. S2C). These results suggest that VacA-dependent AMPK activation is associated with promoting both mitochondrial fragmentation, as well as restoration of mitochondrial function.

### Mitochondrial fission promotes restoration of mitochondrial function in VacA-intoxicated cells

The importance of VacA-dependent AMPK activation for both modulation of mitochondrial dynamics (Fig. 3E; Fig. S8) and restoration of mitochondrial function (Fig. 3B and C; Fig. S5C) prompted us to experimentally evaluate a possible association between mitochondrial fission and restoration of mitochondrial transmembrane potential. These studies revealed that restoration of ΔΨ_m_ (Fig. 4B; Fig. S5C) and cellular ATP levels (Fig. 4C) were impaired in Drp-1-knockdown cells (Fig. 4A; Fig. S5B), which are resistant to fragmentation (60 – 62). These results are consistent with the idea that mitochondrial fission is an important determinant for restoration of mitochondrial function within intoxicated cells.

### Mitochondrial fission is important for limiting VacA accumulation at mitochondria

Our findings thus far indicate that VacA-dependent modulation of mitochondrial dynamics, as well as the capacity of intoxicated cells to limit accumulation of mitochondrial-associated toxin (Fig. 5), both contribute to restoration of mitochondrial function (Fig. 4B C; Fig. S5C). Experiments revealed significantly higher levels of mitochondrial-associated VacA in Drp-1-KD cells than in control cells after 24 hours of exposure to toxin (Fig. 4D; Fig. S7A, B and D). Because restoration of ΔΨ_m_ (Fig. 4B; Fig. S5C) and cellular ATP levels (Fig. 4C) were also impaired in Drp-1-KD cells, these results support the idea that fragmentation of mitochondria through fission is an important step in the restoration of mitochondrial function through a mechanism involving the reduction of mitochondrial-associated VacA.

### Cellular energy sensing and mitochondrial fragmentation are important for the time-dependent decrease in total cellular VacA levels

**Fig 6:**
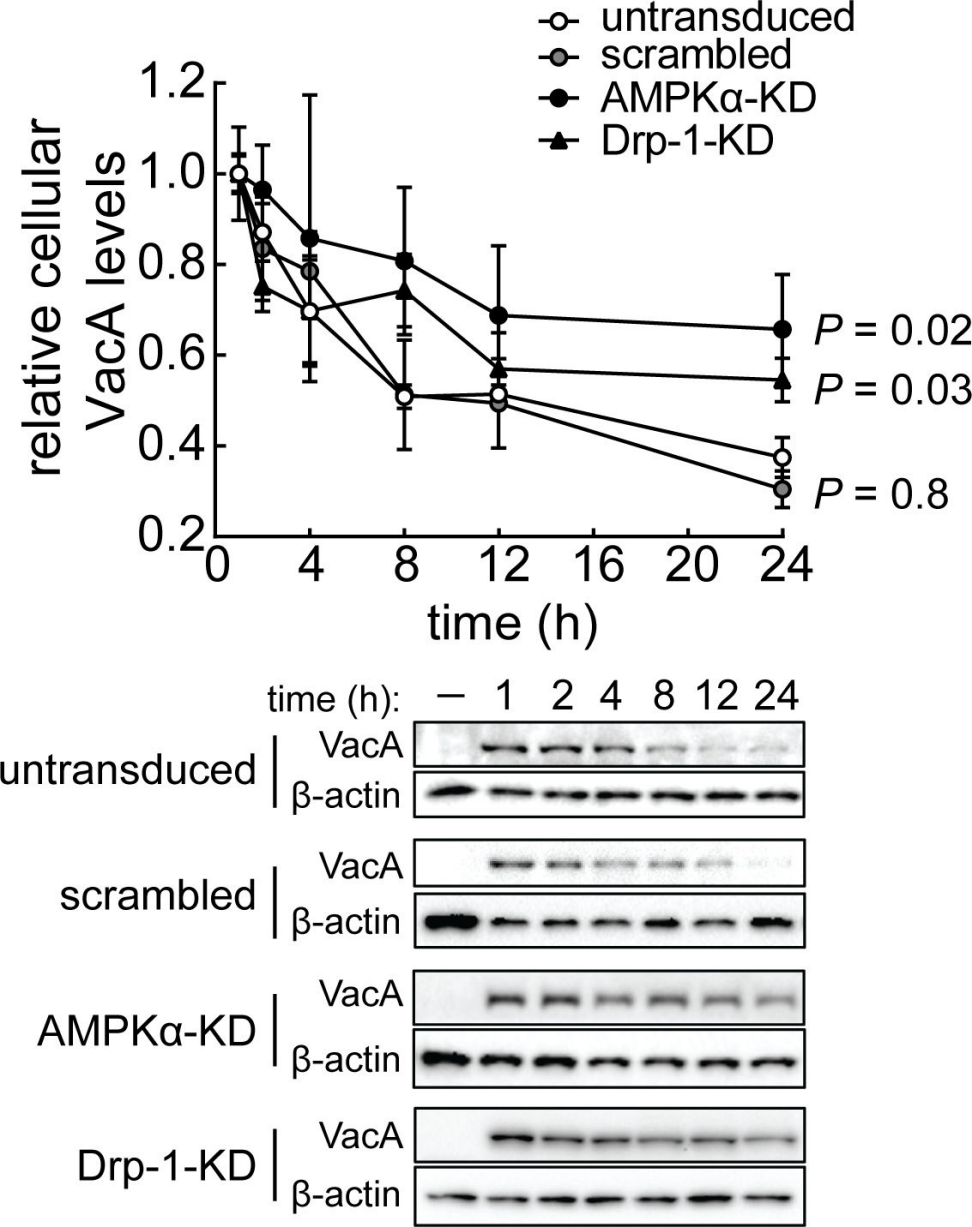
AMPK and Drp-1 are important for time-dependent reduction of total intracellular VacA levels. AZ-521 (untransduced, scrambled, AMPKα-KD, or Drp-1-KD) cells were incubated in the absence or presence of VacA (250 nM) under pulse toxin exposure. Cell lysates were collected and evaluated using immunoblot analysis to determine the relative levels of VacA. The values were relativized to the VacA intensity normalized to β-actin (loading control) at 1 hour. Immunoblots representative of those collected from three independent experiments are shown. Quantification of the band intensities was evaluated by densitometry. The data were combined from three independent experiments (± SD). Statistical significance (α = 0.05) was determined by comparing transduced cells to untransduced cells and was calculated using two-way ANOVA with Tukey’s multiple comparisons test. *P* < 0.05 indicates statistical significance.

We next conducted experiments to evaluate the possibility that reduction in mitochondrial-associated VacA might contribute to the overall time-dependent decline in total intracellular toxin that has been previously reported to occur within intoxicated cells by a lysosomal-dependent mechanism (30). Immunoblot analyses of whole cell lysates to examine changes in toxin levels showed that total intracellular VacA decreased in a time-dependent manner (Fig. 6). Time-dependent reductions in VacA were also detected within non-mitochondrial fractions from lysates of cells that had been exposed to toxin (Fig. S6B and C). Similar to previously reported results (30), inhibition of lysosomal acidification using NH_4_Cl or bafilomycin A1 resulted in increased retention of total intracellular VacA (Fig. S2D and S9), supporting the model that VacA is degraded in a manner dependent upon functional lysosomes. Furthermore, we observed a higher retention of VacA in AMPKα-KD or Drp-1-KD cells (Fig. 6), suggesting that the total intracellular VacA was reduced in a manner associated with both AMPK activation and mitochondrial fission. These results suggest that the reduction in mitochondrial-associated VacA may be linked to and important for overall clearance of intracellular toxin.

### Fission-dependent reduction of mitochondrial-associated VacA promotes viability of intoxicated cells

**Fig 7:**
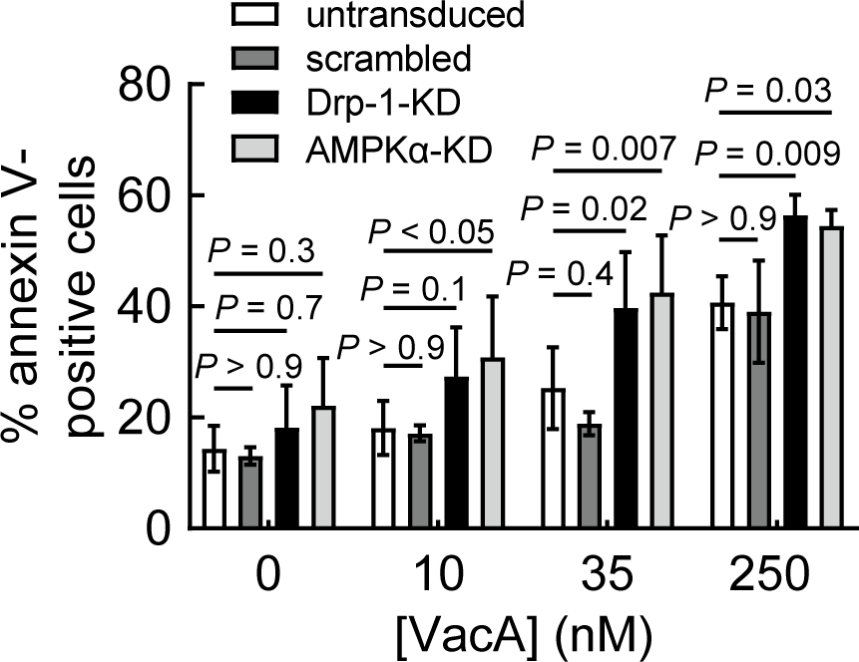
Activation of mitochondrial fission is important for promoting cellular viability within VacA-intoxicated cells. AZ-521 (untransduced, scrambled, AMPKα-KD, or Drp-1-KD) cells were incubated in the absence or presence of VacA (10, 35, 250 nM). After 48 hours, cells were collected and stained with annexin V for analysis by flow cytometry according to the manufacturer’s specifications. The data were combined from three independent experiments (± SD). Statistical significance (α = 0.05) was determined by comparing transduced cells to untransduced cells and was calculated using one-way ANOVA with Tukey’s multiple comparisons test. *P* < 0.05 indicates statistical significance.

Finally, because mitochondrial dysfunction is normally associated with poor cellular health (63, 64), we next assessed the impact of mitochondrial fission on the overall viability of host cells exposed to VacA. In the absence of NH_4_Cl, monolayers of AZ-521 and AGS cells exposed to VacA yielded a modest, but significantly higher percentage of non-viable cells than monolayers not exposed to toxin (Fig. S10). Previously published work reported that, in the presence of NH_4_Cl, AZ-521 cells are more susceptible than AGS cells to VacA-dependent cell death (27, 28). However, our studies, conducted in the absence of NH_4_Cl, revealed higher susceptibility of AGS cells than AZ-521 cells to 250 nM VacA after 48 hours. Notably, these differences in susceptibility between AGS and AZ-521 cells were not observed at lower toxin concentrations (10 and 35 nM) (Fig. S10). Additional experiments revealed a higher percentage of non-viable AMPKα-KD or Drp-1-KD cells than control cells exposed to VacA (Fig. 7). These results support a model that restoration of mitochondrial function, by a mechanism involving cellular modulation of mitochondrial dynamics, is a critical determinant for improving the overall functional vitality of VacA intoxicated cells through restoration of metabolic homeostasis.

## DISCUSSION

**Fig 8:**
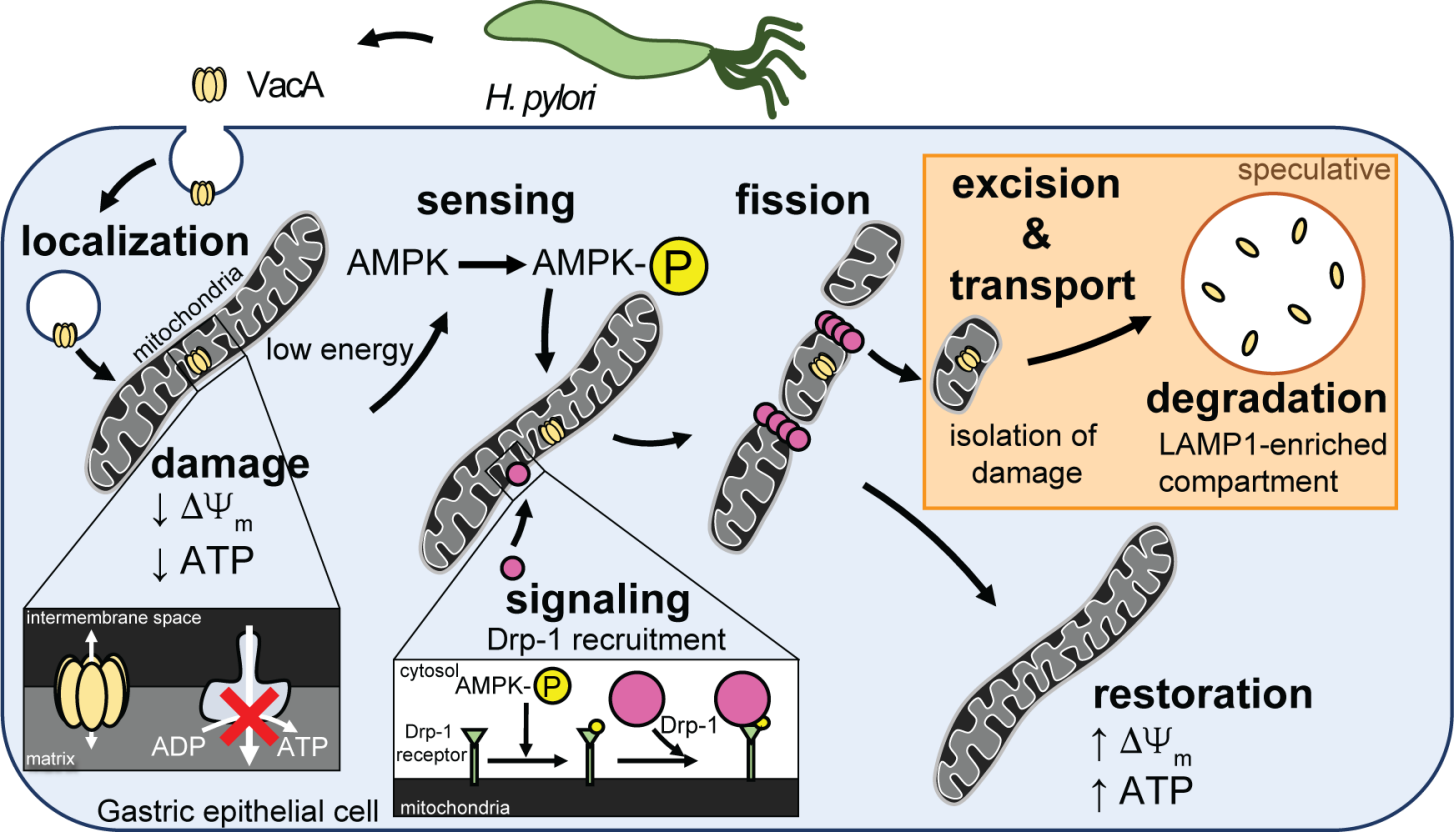
Model describing the cellular mechanism proposed to facilitate mitochondrial restoration during VacA intoxication. VacA is secreted by *Hp* into the extracellular environment and is internalized by gastric epithelial cells. Upon internalization, VacA localizes to mitochondria and forms ion-conducting channels within the inner mitochondrial membrane, thereby inducing mitochondrial dysfunction, characterized by mitochondrial transmembrane depolarization (ΔΨ_m_) and intracellular energy depletion. The depleted energy status is sensed by AMPK, a master energy sensor, resulting in a signaling cascade, which involves AMPK-dependent recruitment of cytosolic Drp-1 to the surface of the mitochondria. Upon recruitment, Drp-1 oligomerizes around the mitochondrial filament and exerts its GTPase activity to induce mitochondrial fission, which is associated with reduction of mitochondrial-associated VacA. We speculate that mitochondrial fission promotes the isolation of mitochondrial lesions containing VacA for transport to a LAMP1-enriched compartment for toxin degradation. We predict that the removal of VacA from mitochondria, when balanced against influx of newly internalized toxin, inhibits accumulation of mitochondrial-associated VacA, which we propose facilitates restoration of mitochondrial function to promote cell viability. The contents framed within the orange box denotes speculative components of our model that remain to be experimentally evaluated.

These studies addressed a poorly understood area of VacA biology, which is the manner by which host cells respond to toxin-dependent targeting and impairment of mitochondrial function during *Helicobacter pylori* infection. The results presented here support a model (Fig. 8) that VacA alterations in mitochondrial function are discerned by AMPK (Fig. 2; Fig. S4), which functions as a central sensor of cellular energy status. Activated AMPK coordinates cellular responses to VacA through the upregulation of mitochondrial fission (Fig. 3E; Fig. S8). Mitochondrial fragmentation was experimentally demonstrated to be important for the restoration of mitochondrial function (Fig. 4B C; Fig. S5C), as well as a reduction in mitochondrial-associated VacA (Fig. 4D). We predict that the removal of VacA from mitochondria, when balanced against influx of newly internalized toxin, limits both the accumulation of VacA at mitochondria and the loss of cell viability (Fig. 7). These findings represent a previously unrecognized strategy by which intoxicated cells sense and curb the mitochondrial damaging effects of pathogenic microbes.

The identification of AMPK activation within VacA intoxicated cells provides important clues into the mechanism underlying cellular responses to toxin targeting of mitochondrial function. As a central cellular sensor of energy status, AMPK operates by integrating stress signals that are indicative of energy shortages into appropriate cellular responses for reestablishing metabolic homeostasis (42, 43, 65). The remarkable ability of activated AMPK to coordinate stress-specific cellular responses is linked to the complex nature of the AMPK assemblage, which comprises three subunits (α, β, γ), each of which has multiple isoforms that assemble in a combinatorial manner, which when coupled with allosteric regulation, fine-tunes AMPK-dependent cellular responses (41, 66 – 68). AMPK activation has been previously reported to be linked to infection with pathogenic microbes that are known to alter cellular metabolism, including *Legionella pneumophila, Neisseria meningitidis,* and *Mycobacterium tuberculosis* (69 – 73). Additionally, bacterial protein toxins, including pore-forming toxins (*Staphylococcus aureus*α-toxin, streptolysin O, *Vibrio cholerae* cytolysin, and *E. coli* hemolysin A), also activate AMPK through indirect damage to mitochondrial function caused by alterations in cellular potassium ion efflux (74). Over the past 20 years, a growing number of pathogens have been identified which generate protein factors that target and localize within mitochondria as part of their virulence strategies (32). However, to the best of our knowledge, VacA is the only protein toxin that induces AMPK activation in a manner dependent on its ability to directly dissipate mitochondrial transmembrane potential.

Our data support a model (Fig. 8) that AMPK activation is dependent upon the pore-forming activity of VacA (Fig. S4D), which is thought to be responsible for depolarization of the inner mitochondrial membrane, resulting in the loss of mitochondrial transmembrane potential and collapse of proton motive force required for ATP production (17, 18, 49, 52). Our data strongly suggest that in response to VacA-dependent reduction in cellular energy, AMPK is activated and then modulates fragmentation of filamentous mitochondria through upregulation of DRP-1-mediated mitochondrial fission. Activated AMPK has been previously demonstrated to modulate mitochondrial structural dynamics through phosphorylation of mitochondrial fission factor, which recruits Drp-1 to the mitochondrial surface for assembly of the mitochondrial fission apparatus (75).

Mitochondrial fragmentation has generally been viewed as a consequence of organelle stress (76, 77). Our finding that Drp-1-dependent mitochondrial fission is important for restoration of mitochondrial function within VacA-intoxicated cells (Fig. 4B C; Fig. S5C) is consistent with experimental evidence linking mitochondrial dynamics to the preservation of mitochondrial function (78, 79). In particular, mitochondrial fission has been identified as an important determinant for the excision of damaged regions from filamentous mitochondrial networks (80). Mitophagy, or mitochondrial-selective autophagy, has been linked to the removal and degradation of large mitochondrial fragments that have been marked as “damaged” (78, 81 – 83), although mitophagy activation in response to VacA intoxication was not detected in our studies (Fig. S3). Alternative mechanisms of mitochondrial quality control have emerged in recent years, including the formation of mitochondrial-derived vesicles (MDVs), which originate from mitochondria, and subsequently fuse with other intracellular compartments, including degradative organelles such as lysosomes (84 – 86). In addition, there is growing evidence that mitochondria physically engage multiple cellular organelles, including the endoplasmic reticulum and lysosomes, through inter-organelle contacts (87 – 90). Although we have not yet investigated these possibilities, we speculate that the removal of mitochondrial-associated VacA through either MDV formation or inter-organelle toxin contacts with lysosomes would constitute an attractive strategy for the selective and localized removal of mitochondrial-associated toxin for degradation without excessive collateral damage to the overall mitochondrial network. In addition, either scenario would be consistent with previous work (30), which was further validated in our studies here (Fig. S9), demonstrating that following exposure to VacA, intracellular levels of toxin decrease by a lysosomal-dependent mechanism.

In conclusion, our results presented here are consistent with the increasing evidence that host cells actively sense the presence of intracellular-acting toxins and effectors through real-time oversight of core cellular activities (91). Indeed, our model predicts that gastric cells possess the capacity to sense the intracellular presence of VacA through detection of toxin activity, and in response, rapidly employ highly specific countermeasures to limit the toxin-mediated dysfunction of mitochondria. Although additional work is required to further understand the cellular mechanism to neutralize the action of VacA, an existing cellular mechanism is likely activated through the detection of the abnormal cellular status, reminiscent of mechanisms described in plant immunity (92). We speculate that toxin-dependent attenuation of host metabolism impairs the capacity of host cells to effectively prevent the establishment of a gastric microenvironment capable of sustaining *Hp* colonization and chronic infection. Finally, in consideration of the highly specific manner that cells respond to intracellular VacA, we anticipate that additional examples of highly specialized host strategies for counteracting the modulatory effects of individual intracellular-acting protein toxins and effectors will continue to be identified, reflecting the complex “give-and-take” that characterizes pathogen-host interactions.

## MATERIALS AND METHODS

### Mammalian cell lines

Mammalian cells were maintained within a humid environment at 37°C and under 5% CO_2_. AZ-521 (human duodenal epithelial) (3940, Riken Japan Health Science Foundation, Wakō, Saitama Prefecture, Japan) and AGS (human gastric epithelial) (CRL-1739, ATCC, Manassas, Virginia) cells were maintained in Dulbecco’s Modification of Eagle’s Medium (DMEM) supplemented with 10% FBS (Sigma-Aldrich, St. Louis, MO) and 1% penicillin/streptomycin (Sigma-Aldrich). Cells were seeded in cell culture plates, dishes, or chamber microscopy slides at cell confluency not exceeding 90%.

### *H. pylori* infection studies

*Hp* 60190 strain (*cag* PAI+, *vacA* s1m1, ATCC 49503), VM022 (*ΔvacA* in 60190) (93), VM084 (*ΔvacA::vacA* in 60190) (94), *Hp vacA* P9A (52), *Hp vacA* G14A (52), *Hp vacA* Δ49–57 (56)*, Hp vacA* Δ346–347 (52, 53)*,* or *Hp vacA* Δ49–57 Δ346–347 (53, 56) strains were inoculated as a suspension in 4 mL bisulfite/sulfite-free *Brucella* (BSFB) broth (0.1% dextrose, 0.2% β-cyclodextrin, 0.5% NaCl [all from Sigma-Aldrich], 1% tryptone, 1% peptone, and 0.2% yeast extract [all from BD Bacto, Franklin Lakes, NJ]) and incubated at 37°C and with humidification on top of 2% agar plates made of Ham’s F-12 media (Sigma-Aldrich) supplemented with 5% fetal bovine serum (FBS) (Sigma-Aldrich), in a microaerophilic environment (10% O_2_, 5% CO_2_). After 2 days, the bacterial cells were harvested by centrifugation at 5,000 × *g* for 1 minute, and the bacterial pellets were suspended in DMEM (10–013-CV, Corning Inc, Corning, NY) supplemented with 10% FBS. AZ-521 cells were infected with *Hp* bacteria (MOI of 10 or 100) at 37°C and under 5% CO_2_ in a humidified environment. To supplement nutrients that may have been depleted by *Hp*, fresh cell culture medium containing 1% penicillin/streptomycin (Sigma-Aldrich) at four times the volume of the initial infection volume was added to the infection 30 minutes prior to endpoint.

### VacA purification

*Hp* 60190 strain (*cag* PAI^+^, *vacA* s1m1, ATCC49503) was cultured under biphasic conditions, with Ham’s F-12 agar (supplemented with 10% fetal bovine serum and 5 µg/mL vancomycin) and BSFB for 48 hours at 37°C under microaerophilic conditions (10% O_2_, 5% CO_2_). The cell suspension collected as a starter culture inoculum was diluted 100-fold in fresh BSFB, which was further cultured for 48 hours at 37°C and under 5% CO_2_. After incubation, culture supernatant was collected by centrifuging *Hp* bacterial cultures at 10,000 × *g* for 20 minutes at 4°C and precipitated using 662 g/L ammonium sulfate (Fisher Scientific, Waltham, MA) overnight at 4°C with rotation. Precipitated proteins were collected by centrifuging the mixture at 10,000 × *g* for 20 minutes at 4°C and resuspended with a phosphate buffer [10 mM Na_2_HPO_4_ (Sigma), pH7]. The resuspended protein suspension was twice dialyzed in 50K MWCO dialysis membrane (Spectra/Por 6 RC, Spectrum Chemical, New Brunswick, NJ) in phosphate buffer at ~150 times the volume over the course of 24 hours. The dialyzed protein suspension was centrifuged at 10,000 × *g* for 5 minutes at 4°C and filter-sterilized through 0.22 µm pore PES filters (Millipore, Burlington, MA) and loaded on a diethylaminoethyl (DEAE)-sephacel anion exchange column (GE17-0500-01, Sigma-Aldrich) equilibrated in phosphate buffer. After washing off unbound proteins using two bed volumes of phosphate buffer, proteins were eluted using the elution buffer [10 mM Na_2_HPO_4_ (Sigma), 200mM NaCl, pH7]. The collected fractions were evaluated by SDS-PAGE and Coomassie staining for the presence of pure VacA, characterized by the molecular weight of 88 kDa making up >95% of total lane bands. VacA-positive eluate was loaded into 50 kDa MWCO Amicon Ultra Centrifugal Filter (UFC905024, Millipore) using PBS (pH 7.4) 10 times the total eluate volume. VacA was quantified using Coomassie Plus Bradford Assay Kit according to the manufacturer’s instruction (23238, Thermo Fisher Scientific).

### Acid activation of VacA

Before introducing to cells, VacA was activated by mixing VacA with 0.3 M HCl at 10:1 ratio and incubating at 37°C for 30 minutes. After incubation, 0.3 M NaOH equal volume as 0.3 M HCl was added to neutralize the solution. For the negative control, sterile PBS (pH 7.4) was used in place of VacA. Activated VacA was diluted in cell culture media, exceeding no more than 10% of the total volume and was used immediately to treat cells.

### Immunoblot analyses

Cell lysates were prepared by incubating monolayers in lysis buffer (20 mM Tris pH 7.5, 100 uM Na3VO4, 25 mM NAF, 25 mM β-glycerolphosphate, 2 mM EGTA, 2 mM EDTA, 0.3% Triton X-100; Sigma-Aldrich) that was supplemented with cOmplete Mini EDTA-free protease inhibitor cocktail tablets (1 tablet/25 mL, 11836170001, Roche Diagnostics, Indianapolis, IN). After 10 minutes, the lysates were transferred to fresh tubes and mixed with an equivalent volume of 2× sodium dodecyl sulfate-polyacrylamide gel electrophoresis (SDS-PAGE) buffer (100 mM, SDS Tris pH 6.8, 4% bromophenol blue, 0.2% glycerol, 20% 2-mercaptoethanol; Sigma-Aldrich), and incubated at 100°C for 10 minutes. After SDS-PAGE, the gel proteins were electro-transferred to a polyvinylidene fluoride (PVDF) membrane (Millipore). The transfer membranes were incubated overnight at 4°C with primary antibody diluted in blocking buffer [Tris-buffered pH 7.4 saline with 0.1% Tween 20 (TBS-T)] supplemented with 5% skim milk (Lab Scientific, Highlands, NJ). The blots were briefly washed for 5 minutes with shaking and incubated for 1 hour at room temperature with HRP-conjugated secondary antibody (1:5,000 dilution, Cell Signaling Technology, Danvers, MA) diluted with TBS-T. After washing four times with TBS-T for 5 minutes each, the blots were incubated with HRP substrates [SuperSignal West, Pico (34580) and Femto (34095) mixed at 1:1 ratio, Thermo Fisher Scientific]. The luminescent signals from the blots were imaged using a ChemiDoc XRS + imaging system (Bio-Rad, Hercules, CA) and analyzed using Image Lab software (Version 6.0; Bio-Rad) to quantify the relative band intensities.

### Antibodies and small molecule inhibitors

The following antibodies were used in this paper: β-actin (cs-4970, Cell Signaling Technology), TOM20 (sc-17764, Santa Cruz Biotechnology, Dallas, TX), TIM23 (BD-611222, BD Biosciences, Franklin Lakes, NJ), VacA (19), phospho-ACC (11818, Cell Signaling Technology), ACC (3676, Cell Signaling Technology), AMPKα (2532, Cell Signaling Technology), DRP1 (8570, Cell Signaling Technology), LAMP1 (9091, Cell Signaling Technology), Calnexin (2679, Cell Signaling Technology), GAPDH (5174, Cell Signaling Technology), Syntaxin 6 (2869, Cell Signaling Technology), VDAC (4866, Cell Signaling Technology), Anti-rabbit IgG HRP-linked (7074, Cell Signaling Technology), Anti-mouse IgG HRP-linked (7076, Cell Signaling Technology), Alexa Fluor 555-conjugated anti-mouse antibody (A31570, Thermo Fisher Scientific), and Alexa Fluor 488-conjugated anti-rabbit antibody (A21206, Thermo Fisher Scientific). The following small molecules were used in this paper: CCCP (C2759, Sigma), Compound C (171260, Sigma-Aldrich), and Bafilomycin A1 (196000, Sigma-Aldrich).

### Limited VacA exposure (pulse)

Monolayers of cells were incubated in the absence or presence of VacA at the indicated concentrations in prewarmed cell culture medium at 37°C and under 5% CO_2_ for 10 minutes. After 10 minutes, cells were washed twice in PBS and incubated in prewarmed cell culture medium in the absence of VacA for the remainder of the indicated duration at 37°C under 5% CO_2_.

### Measurement of mitochondrial transmembrane potential

Mitochondrial transmembrane potential was determined by incubating cells in cell culture medium in the presence of 10 nM TMRE (T669, Invitrogen, Waltham, MA) for 30 minutes or 50 nM TMRE for 10 minutes prior to collection. Cells were exposed to 50 nM TMRE for 10 minutes for the time course studies to accommodate the short incubation periods. After incubation, cells were washed in PBS, detached using 0.05% trypsin (Gibco, Billings, Montana), and collected in cold 10% FBS diluted in PBS. TMRE fluorescence intensity was measured by flow cytometry using the PE channel with bandpass filter of 585/42 nm on a BD FACSCanto II CMtO Analyzer (BD Biosciences). For each replicate, at least 10,000 events were collected. Data combined from three independent experiments were relativized to the unintoxicated control for each cell variant.

### Measurement of intracellular ATP levels

Cells were seeded on a 96-well tissue culture-treated plate (flat bottom, white with clear bottom) (3903, Corning Inc) to achieve 50%–70% confluency prior to the beginning of the assay. After completion of the treatment, intracellular ATP levels were assessed using the Luminescent ATP Detection Assay Kit (ab113849, Abcam, Cambridge, United Kingdom) according to the manufacturer’s protocol. Using a Synergy 2 plate reader (BioTek, Winooski, VT), luminescence was measured after 10 minutes in the dark. The intracellular ATP levels were normalized to total protein determined by the BCA assay (Thermal Fisher) according to the manufacturer’s instruction. The normalized luminescence values of intoxicated cells were compared relative to unintoxicated cells. Data were collected from three independent replicates each performed with at least two technical replicates.

### Analysis of AMPK activities

The relative AMPK activities within whole cell lysates was determined by immunoblot analysis using p-ACC (S79) antibody (1:1,000 dilution), ACC antibody (1:1,000 dilution), or β-actin antibody (1:5,000 dilution) calculated from the intensity of the immuno-specific signal corresponding to p-ACC divided by the intensity of the band corresponding to total ACC. The immunofluorescence microscopy analysis of AMPK activities was performed using p-ACC (S79) antibody.

### Immunofluorescence microscopy

Mammalian cells were seeded in 8-well chamber slides (Thermo Fisher Scientific) and were incubated in the absence or presence of VacA (continuous or pulse exposure) in cell culture medium at 37°C and 5% CO_2_. After completion of treatment, cells were fixed in 4% paraformaldehyde in PBS (pH 7.4) for 15 minutes, were permeabilized using 0.1% Triton X-100 in PBS (pH 7.4) for 15 minutes, and were incubated in blocking solution (5% BSA and BP1600-100 [Fisher Scientific] in PBS [pH 7.4]) for 1 hour. Primary and fluorophore-conjugated secondary antibodies were diluted in 0.1% Tween-20 in PBS (pH 7.4) and incubated overnight at 4°C and 2 hour at room temperature, respectively. Cells were then incubated with 0.5 µg/mL DAPI in PBS (pH 7.4) for 15 minutes. After washing cells three times using 0.1% Tween-20 diluted in PBS (pH 7.4), cells were mounted using Prolong Gold Antifade (P36930, Invitrogen) with a coverslip. Mounted cells were observed using Zeiss LSM 700 confocal microscope system with a Zeiss AXIO Observer Z1 inverted microscope stand. The images were processed and analyzed using Zen Black 2012.

### Generation of knockdown via shRNA

Lentiviral particles used for transduction were generated in HEK293T cells that were co-transfected with 0.5 µg/mL shRNA-carrying plasmid, 0.45 µg/mL packaging vector pCMV-dR8.2 (plasmid# 8455, Addgene, Watertown, MA), and 0.05 µg/mL enveloping vector pCMV-VSV-G (plasmid# 8454, Addgene) using Lipofectamine 2000 (11668019, Thermo Fisher Scientific). Lentiviral clones expressing shRNA against human PRKAA1 (AMPKα1) (TRCN0000196482, Sigma-Aldrich), human PRKAA2 (AMPKα2) (TRCN0000002171, Sigma-Aldrich), and human Drp-1 shRNA (sc-43732-SH, Santa Cruz Biotechnology) were used for generating lentiviral particles. After 48 hours, medium containing the viruses were collected and used to infect AZ-521 or AGS cells in the presence of 8 µg/mL polybrene. Control scramble shRNA Lentiviral Particles-A (sc-108080, Santa Cruz Biotechnology), containing shRNA sequence that does not lead to degradation of known cellular mRNA in mammalian cells, was used to generate negative control. After 24 hours, cells were subjected to selection using 2 µg/mL puromycin for 96 hours. Efficiency of knockdown was determined via immunoblot analysis.

### Quantification of mitochondrial-associated VacA levels

Mitochondria were isolated from cells incubated in the absence or presence of VacA using the Mitochondria Isolation Kit for Cultured Cells (89874, Thermo Fisher) according to the manufacturer’s instructions. The cell lysate and non-mitochondrial fractions were prepared by adding 6× SDS loading dye (786–701, G Biosciences, St. Louis, MO) supplemented with Halt Protease & Phosphatase Single-Use Inhibitor Cocktail (78442, Thermo Scientific). The purity of mitochondrial fractions was confirmed by immunoblot analysis of the WCL, supernatant, and mitochondrial fractions that were normalized by volume using antibodies against TIM23 (1:1,000 dilution), LAMP1 (1:1,000 dilution), Calnexin (1:1,000 dilution), GAPDH (1:5,000 dilution), and Syntaxin 6 (1:1,000 dilution). Mitochondrial-associated VacA levels were determined through immunoblot analysis, calculated from the intensity of the band corresponding to VacA (1:100,000 dilution) and normalized from the intensity of the band corresponding to the mitochondrial loading control, TIM23 (1:1,000 dilution).

### Mitochondrial structural analysis

Mitochondrial structure was detected using immunofluorescence by incubating fixed, permeabilized, and blocked cells at 4°C with TOM20 antibody (1:1,000 dilution) diluted in 1% BSA (Fisher Scientific) in PBS (pH 7.4). After an overnight incubation, cells were washed with 0.1% Tween-20 diluted in PBS (pH 7.4), followed by incubation for 2 hours in the dark at room temperature with donkey Alexa Fluor 555-conjugated anti-mouse antibody (1:1,000 dilution, Thermo Fisher Scientific, A31570). The percentage of cells displaying predominantly fragmented mitochondria was calculated by dividing the number of cells exhibiting predominantly fragmented mitochondria scattered throughout the cell (with no obvious filamentous mitochondrial structure within the cell) by the number of total cells in the field. Rounded cells were excluded from quantification. For each experimental condition, at least five visual fields, containing at least two cells per field, were assessed for three independent experiments. Scoring of the images was conducted in a blinded manner, in which the evaluator was not informed of the treatment conditions.

### Assessing cellular VacA levels

After completion of treatment, cell lysates were collected and subjected to immunoblot analysis using methods described above. The cellular levels of VacA were determined by immunoblot analysis using rabbit VacA (1:100,000 dilution), normalized to the load control, β-actin (1:5,000 dilution).

### Cell death assay

Monolayers of cells were incubated in the absence or presence of VacA at 37°C under 5% CO_2_. After 48 hours, cells and supernatant were collected and stained with Annexin V-Alexa Fluor 488, as described by the manufacturer (V13241, Thermo Fisher Scientific). Fluorescence intensity was measured by flow cytometry using a bandpass filter of 530/30 nm and 655/LP-nm with compensation on a BD FACSCanto II flow analyzer (BD Biosciences).

### Statistical analysis

Each experiment was performed at least three independent times (*n* = 3). Statistical analyses were conducted using GraphPad Prism (v. 8.4.3). Error bars represent the standard deviations. *P*-values shown were calculated using the indicated tests (unpaired *t*-test, one-way or two-way ANOVA with Tukey’s, Sidak’s, or Dunnett’s multiple comparisons test). *P*-values less than 0.05 (α = 0.05) were considered statistically significant.

## SUPPLEMENTAL MATERIAL

Fig. S1 mbio.02117-23-s0001.tif VacA-mediated mitochondrial dysfunction is restored in a time- and concentration-dependent manner in AGS cells.

Fig. S2 mbio.02117-23-s0002.tif Effects of NH4Cl on VacA intoxicated cells.

Fig. S3 mbio.02117-23-s0003.tif Mitochondrial mass after VacA intoxication or treatment with mitophagy-inducing agents.

Fig. S4 mbio.02117-23-s0004.tif VacA-mediated AMPK activation.

Fig. S5 mbio.02117-23-s0005.tif Mitochondrial transmembrane potential recovery in AGS AMPKα- and Drp-1-knockdown cells.

Fig. S6 mbio.02117-23-s0006.tif VacA cellular and subcellular localization.

Fig. S7 mbio.02117-23-s0007.tif Immunoblot analysis of time-dependent mitochondrial-associated VacA levels.

Fig. S8 mbio.02117-23-s0008.tif VacA-mediated mitochondrial fragmentation is blocked in the presence of Compound C.

Supplemental legends mbio.02117-23-s0009.docx Legends for supplemental figures.

Fig. S9 and Fig. S10 mbio.02117-23-s0010.tif Cellular VacA levels are protected from lysosomal degradation in the presence of bafilomycin A1. Comparison of VacA-mediated cell death in AZ-521 and AGS cells.

ASM does not own the copyrights to Supplemental Material that may be linked to, or accessed through, an article. The authors have granted ASM a non-exclusive, world-wide license to publish the Supplemental Material files. Please contact the corresponding author directly for reuse.
