# Supplementary figures and images for: Host cell sensing and restoration of mitochondrial function and metabolism within Helicobacter pylori VacA intoxicated cells
Source: mBio. 2023 Oct 10;14(5):e02117-23. doi: 10.1128/mbio.02117-23 (PMC10653863; doi:10.1128/mbio.02117-23)

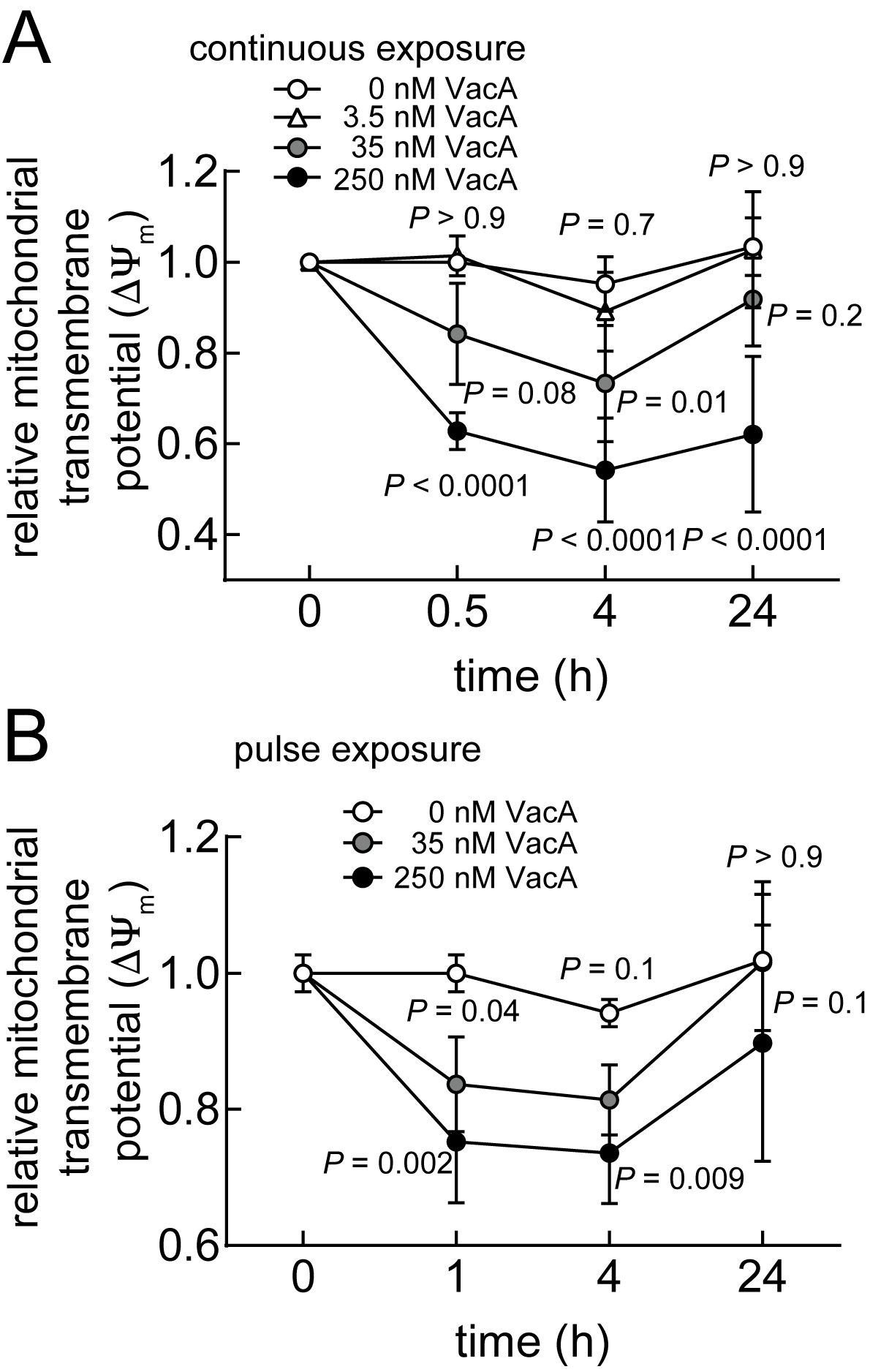

Supplement: Fig. S1 — VacA-mediated mitochondrial dysfunction is restored in a time- and concentration-dependent manner in AGS cells. [file mbio.02117-23-s0001.tif]

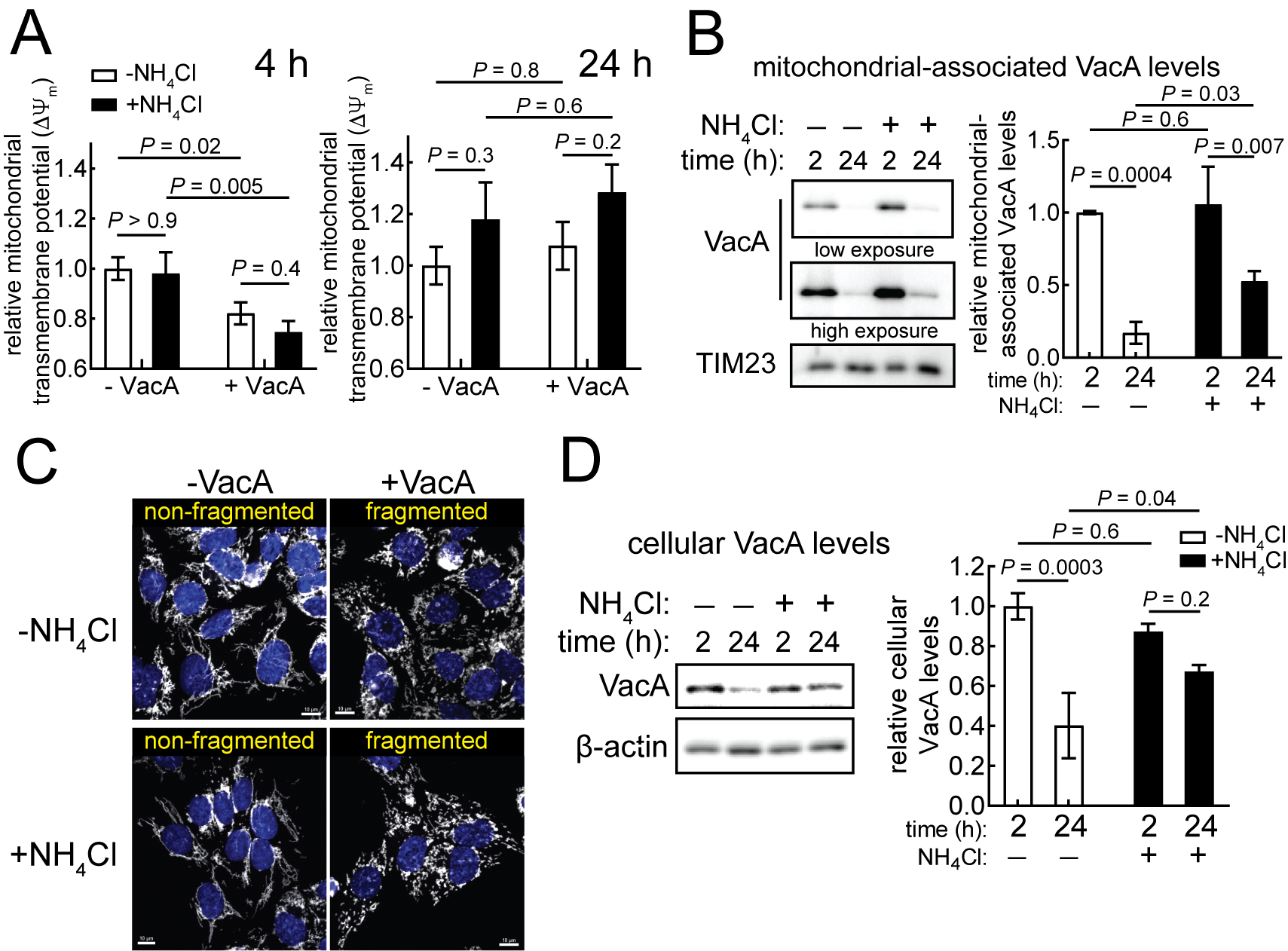

Supplement: Fig. S2 — Effects of NH4Cl on VacA intoxicated cells. [file mbio.02117-23-s0002.tif]

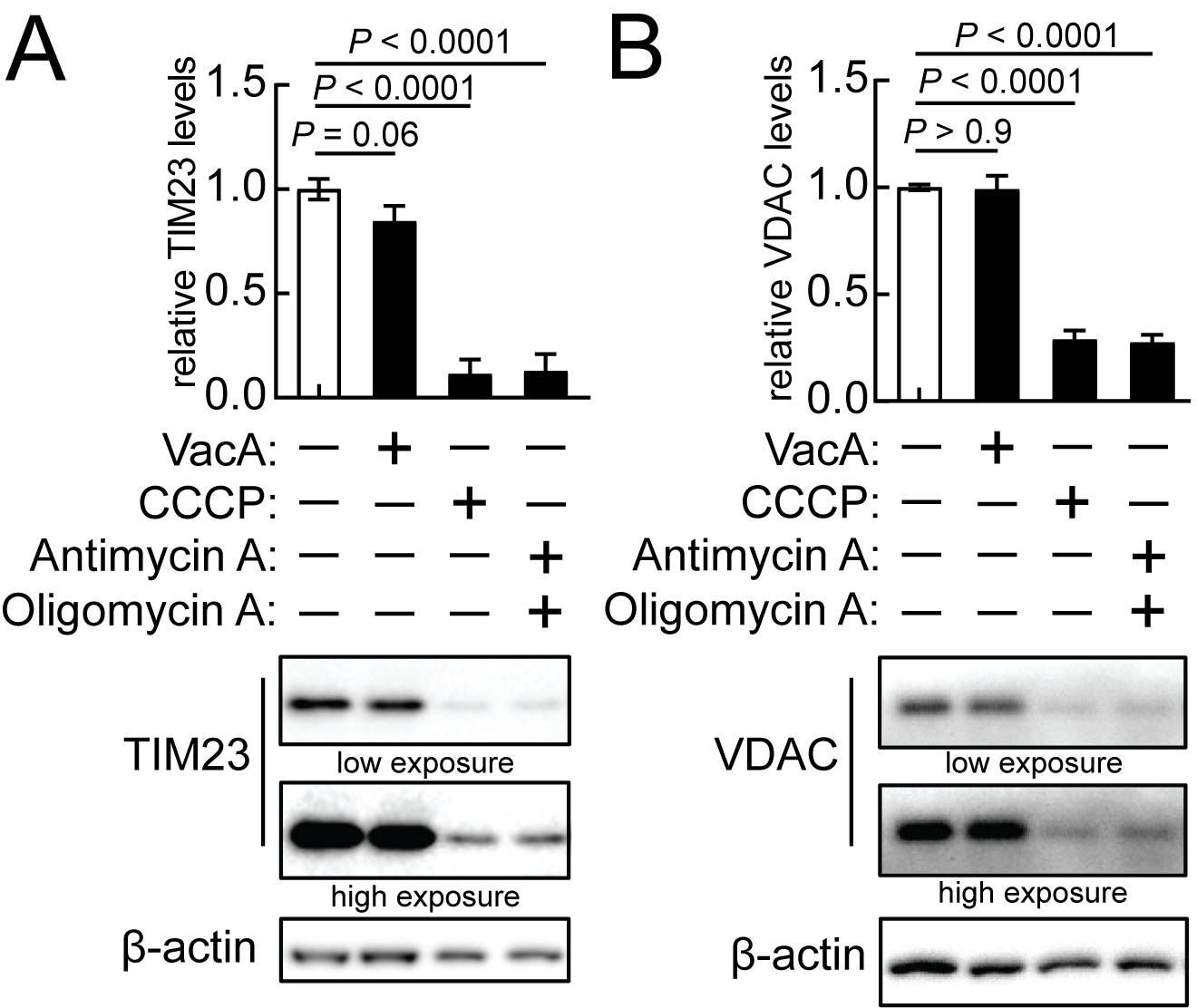

Supplement: Fig. S3 — Mitochondrial mass after VacA intoxication or treatment with mitophagy-inducing agents. [file mbio.02117-23-s0003.tif]

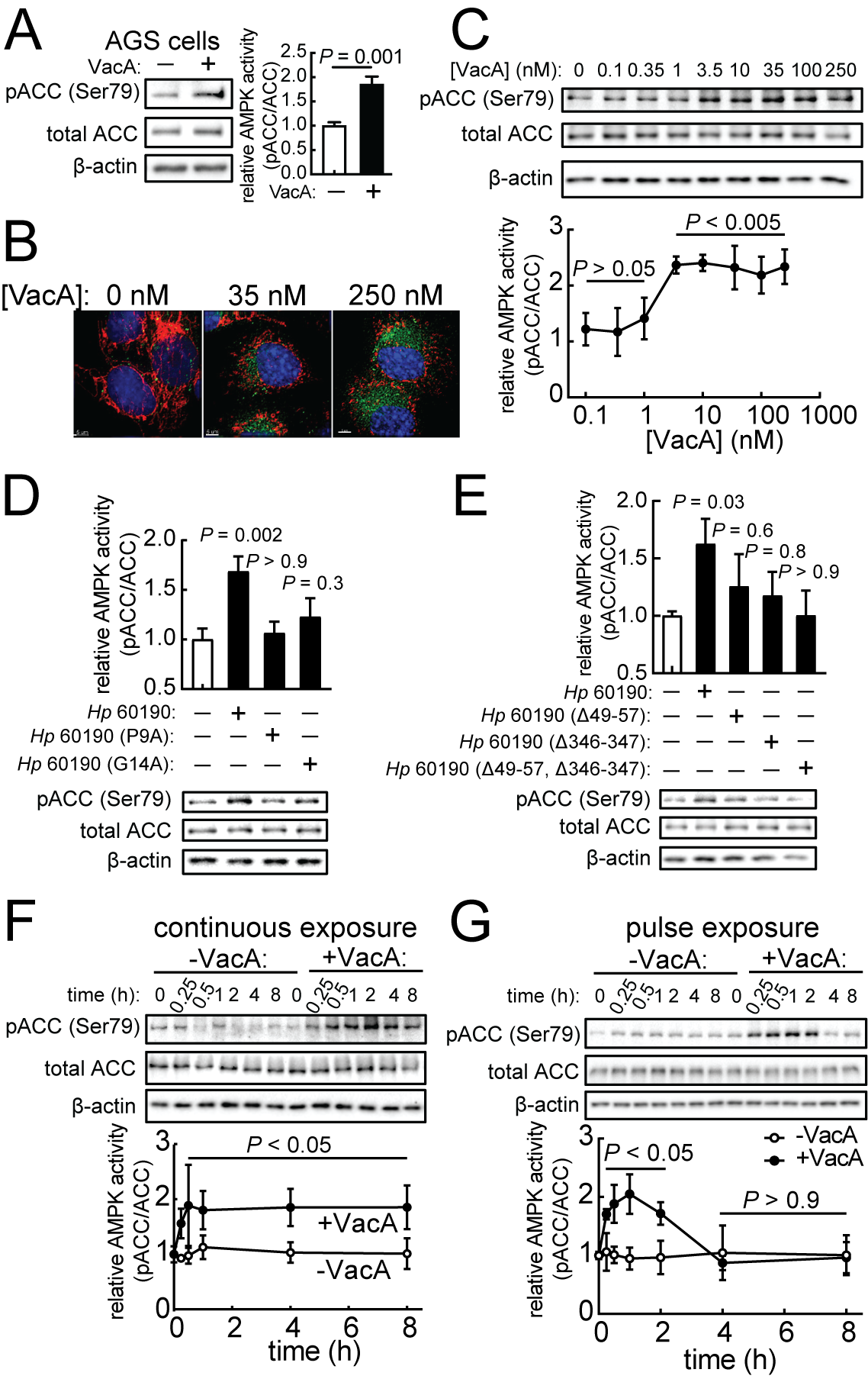

Supplement: Fig. S4 — VacA-mediated AMPK activation. [file mbio.02117-23-s0004.tif]

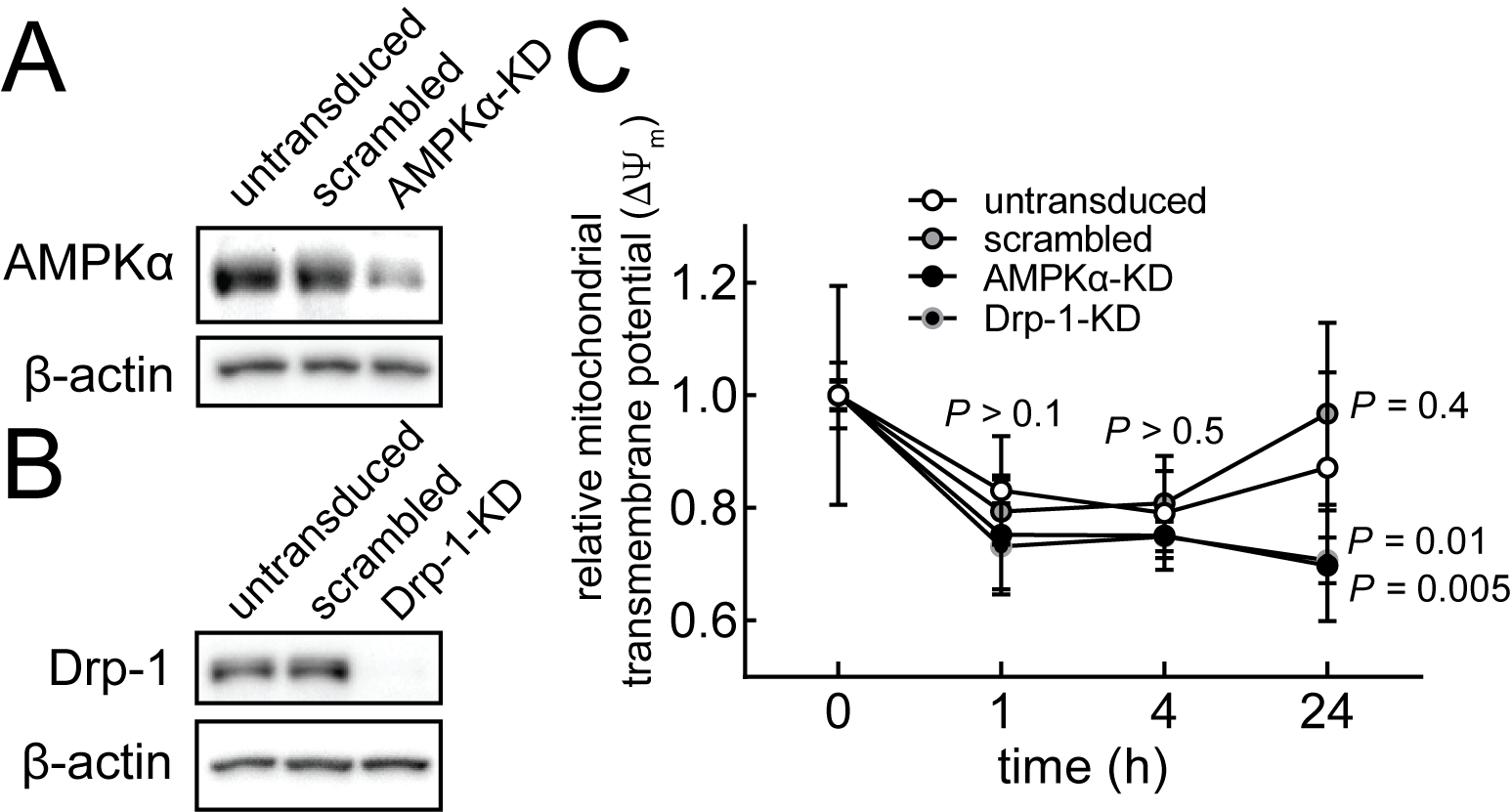

Supplement: Fig. S5 — Mitochondrial transmembrane potential recovery in AGS AMPKα- and Drp-1-knockdown cells. [file mbio.02117-23-s0005.tif]

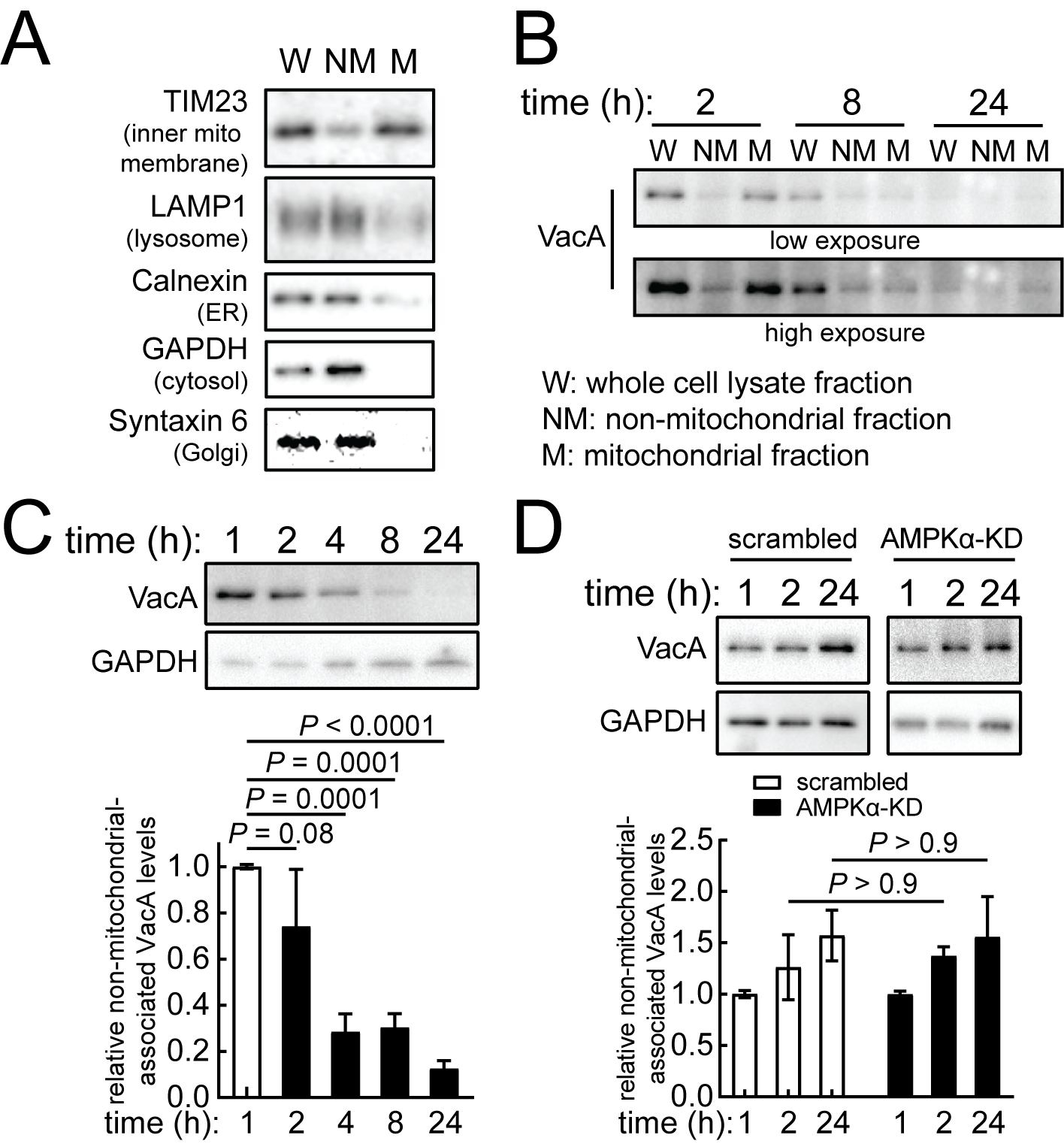

Supplement: Fig. S6 — VacA cellular and subcellular localization. [file mbio.02117-23-s0006.tif]

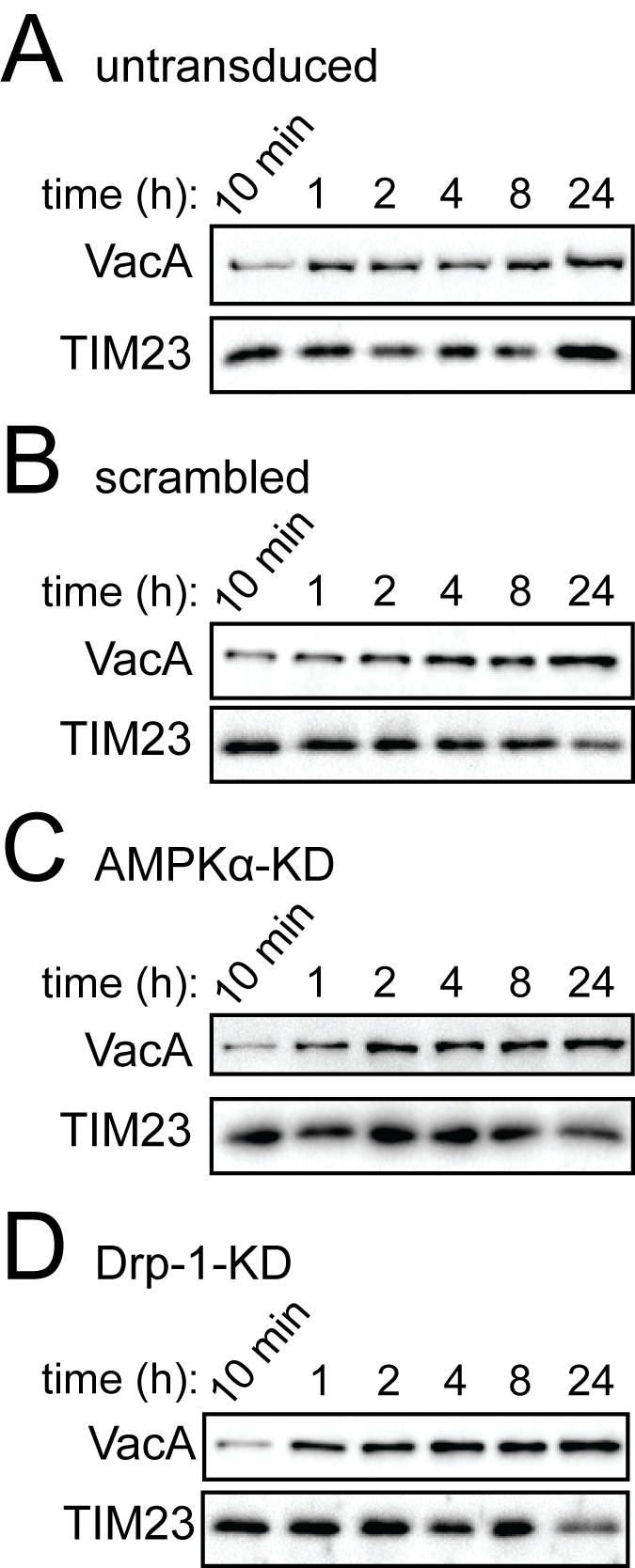

Supplement: Fig. S7 — Immunoblot analysis of time-dependent mitochondrial-associated VacA levels. [file mbio.02117-23-s0007.tif]

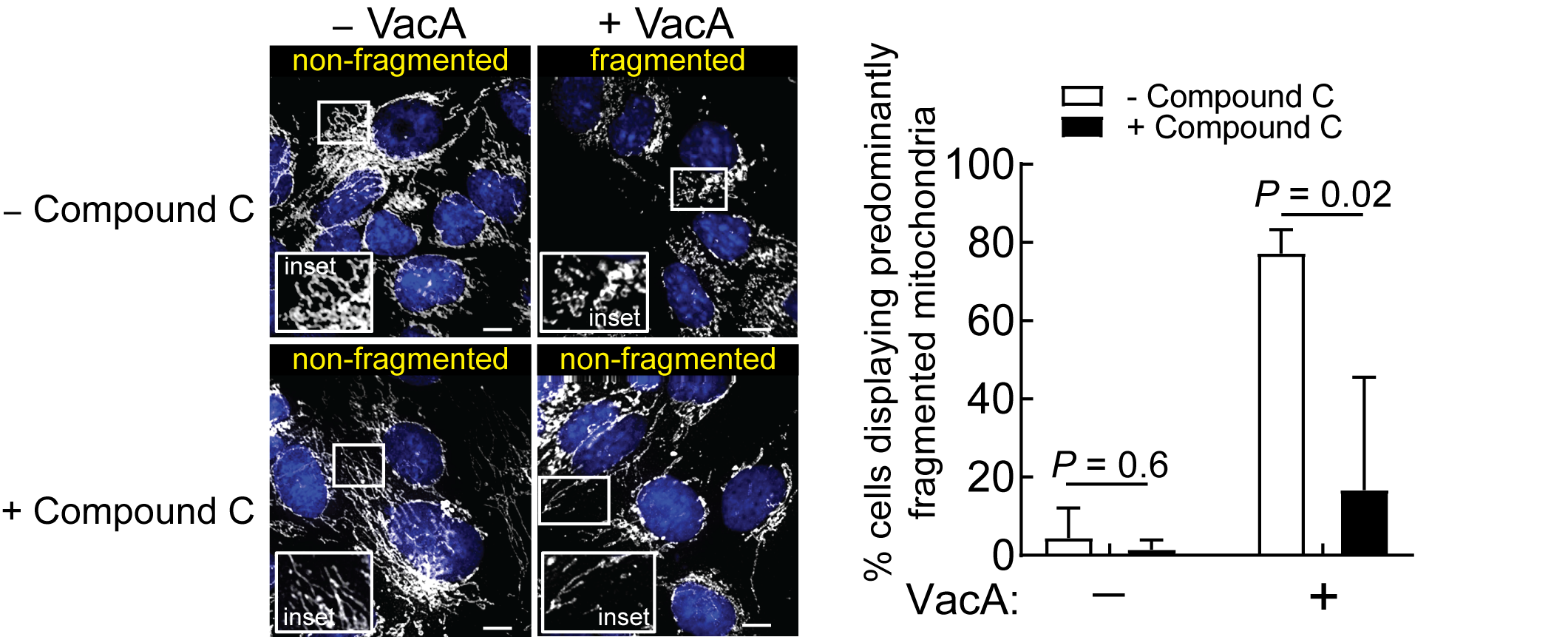

Supplement: Fig. S8 — VacA-mediated mitochondrial fragmentation is blocked in the presence of Compound C. [file mbio.02117-23-s0008.tif]

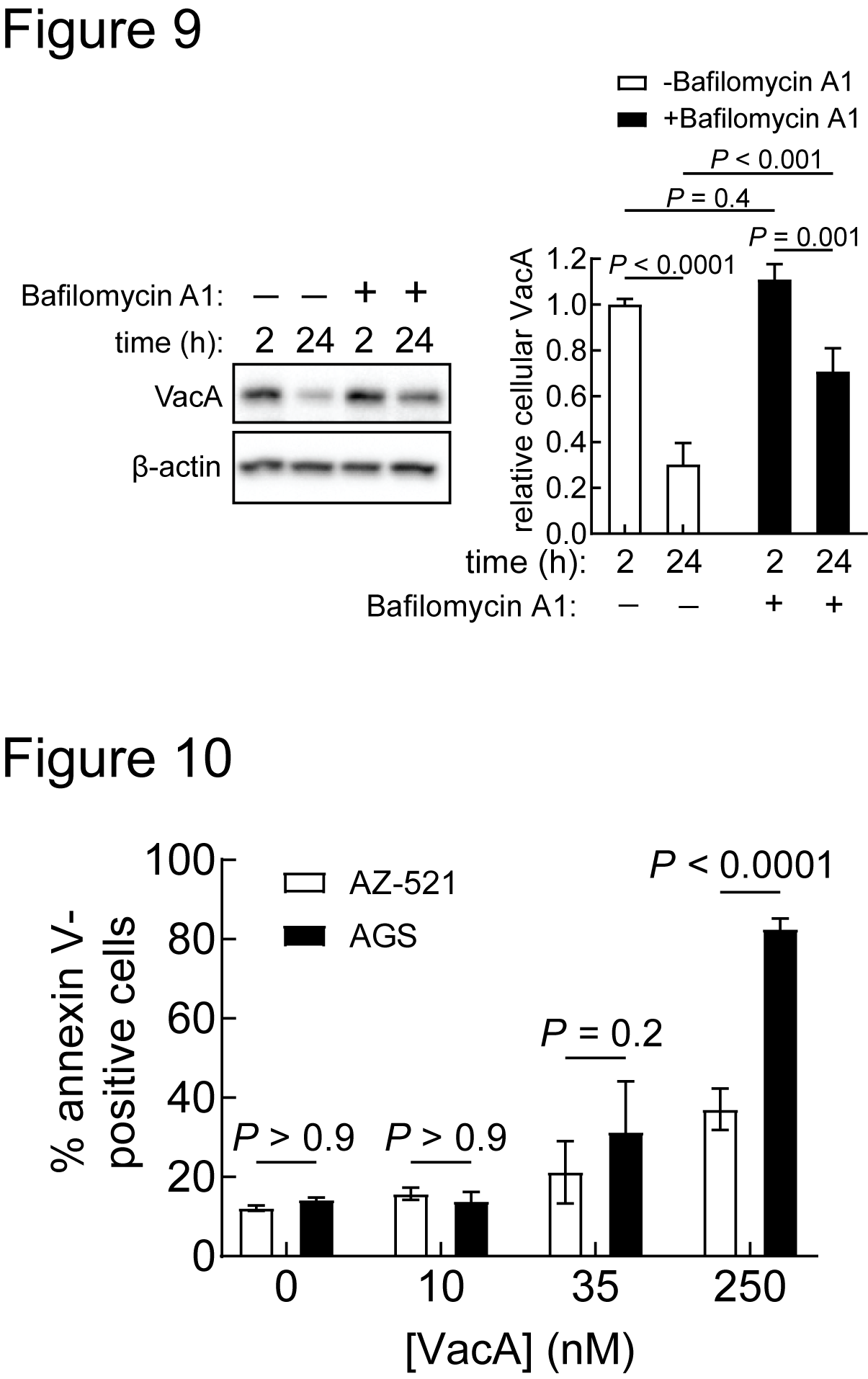

Supplement: Fig. S9 and Fig. S10 — Cellular VacA levels are protected from lysosomal degradation in the presence of bafilomycin A1. Comparison of VacA-mediated cell death in AZ-521 and AGS cells. [file mbio.02117-23-s0010.tif]
