# Supplementary material for: Host cell sensing and restoration of mitochondrial function and metabolism within Helicobacter pylori VacA intoxicated cells
Source: mBio. 2023 Oct 10;14(5):e02117-23. doi: 10.1128/mbio.02117-23 (PMC10653863; doi:10.1128/mbio.02117-23)
Supplement: Supplemental legends — Legends for supplemental figures. [file mbio.02117-23-s0009.docx]

**Fig S1: VacA-mediated mitochondrial dysfunction is restored in a time- and concentration-dependent manner in AGS cells.**

AGS cells were incubated in the absence or presence of VacA (3.5, 35, 250 nM), which we have defined as “continuous exposure” of cells to VacA (A). Alternatively, AGS cells were incubated for 10 min in the absence or presence of VacA (35 or 250 nM), which we have defined as “pulse exposure” of cells to VacA. After 10 min, cells were twice washed with PBS (pH 7.4) to eliminate any unbound extracellular VacA and further incubated in the presence of fresh cell culture medium for the duration of the experiment (B). 10 (A) or 30 (B) min prior to cell collection, cells were stained with TMRE. Stained cells were evaluated for mitochondrial transmembrane potential (ΔΨ_m_) using flow cytometry. The fluorescence values of intoxicated cells were compared relative to unintoxicated cells. The data were combined from three independent experiments (± SD). Statistical significance (α = 0.05) was calculated using two-way ANOVA with Dunnett’s multiple comparisons test. *P* < 0.05 indicates statistical significance.

**Fig S2: Effects of NH_4_Cl on VacA intoxicated cells.**

AZ-521 cells were incubated in the presence or absence of 5 mM NH_4_Cl. After 30 min, cells were incubated in the presence of VacA (250 nM) for pulse (A, B, and D) or continuous (C) toxin exposure in the presence or absence of 5 mM NH_4_Cl. To evaluate mitochondrial functional restoration (A), cells were stained with 10 nM TMRE and evaluated for mitochondrial transmembrane potential (ΔΨ_m_) using flow cytometry. The fluorescence values of intoxicated cells were compared relative to unintoxicated cells. To evaluate for mitochondrial-associated (B) or total cellular (D) VacA levels, whole cell lysates (B) or mitochondrial fractions (D) were collected and processed for immunoblot analysis. Quantification of the band intensities was evaluated by densitometry, and the values were compared relative to the VacA levels from untreated cells at 2 h. VacA was not detected in mitochondrial fractions from cells incubated in the absence of VacA. The data were combined from three independent experiments (± SD). Statistical significance (α = 0.05) was calculated by two-way ANOVA with Sidak’s multiple comparisons test. *P* < 0.05 indicates statistical significance. To evaluate mitochondrial structure, cells were fixed after 1 h and evaluated by fluorescence microscopy, using TOM20 as a marker for mitochondrial structure (C). Fluorescence images (White = TOM20; Blue = DAPI) shown are representative of multiple fields. Scale bars indicate 10 µm.

**Fig S3: Mitochondrial mass after VacA intoxication or treatment with mitophagy-inducing agents.**

To evaluate mitochondrial mass, AZ-521 cells were incubated in the absence or presence of VacA (250 nM), CCCP (50 µM), or a combination of Oligomycin A (10 µM) and Antimycin A (50 µM). After 24 h, cell lysates were collected and evaluated using immunoblot analysis to determine the relative levels of TIM23 (A; inner mitochondrial membrane) and VDAC (B; outer mitochondrial membrane) as surrogates for mitochondrial mass. The values shown were normalized to β-actin (loading control) and are shown relative to untreated cells. Immunoblots representative of those collected from three independent experiments are shown. Quantification of the band intensities was evaluated by densitometry. The data were combined from three independent experiments (± SD). Statistical significance (α = 0.05) was calculated using one-way ANOVA with Dunnett’s multiple comparison’s test. *P* < 0.05 indicates statistical significance.

**Fig S4: VacA-mediated AMPK activation.**

AGS cells were incubated in the absence or presence of VacA (250 nM) for 1 h (A). AZ-521 cells were incubated in the absence or presence of VacA (35 or 250 nM) (B) or a dose range of VacA (0.1, 0.35, 1, 3.5, 10, 35, 100, 250 nM) for 1 h (C). Alternatively, AZ-521 cells were infected with *Hp* 60190 (D and E), *Hp* 60190 (P9A), *Hp* 60190 (G14A) (D), *Hp* 60190 (Δ49-57), *Hp* 60190 (Δ346-347), or *Hp* 60190 (Δ49-57Δ346-347) (E) at MOI 100 for 2 h. AZ-521 cells were also incubated in the absence or presence of VacA (250 nM; for 0, 0.25, 0.5, 1, 2, 4, 8 h) under continuous (F) or pulse (G) toxin exposures. Cell lysates were collected and evaluated using immunoblot analysis (A, and C to G), or immunofluorescence imaging (B), to determine the relative levels of phospho-ACC (S79) (pACC (S79)). Immunoblots representative of those collected from three independent experiments are shown. Quantification of the band intensities was evaluated by densitometry. The data were combined from three independent experiments (± SD) (A, and C to G). Images are representative of three independent biological replicates (B). Scale bar indicates 5 µm. Green: phospho-ACC (Ser79), Red: TOM20 (mitochondria), Blue: DAPI. Statistical significance (α = 0.05) was calculated using an unpaired *t*-test (A), one-way ANOVA with Dunnett’s multiple comparisons test (C), one-way ANOVA with Tukey’s multiple comparisons test (D and E), or two-way ANOVA with Sidak’s multiple comparisons test (F and G). *P* < 0.05 indicates statistical significance.

**Fig S5: Mitochondrial transmembrane potential recovery in AGS AMPKα- and Drp-1-knockdown cells.**

AGS AMPKα-KD cells and AGS Drp-1-KD cells were generated and confirmed using immunoblot analysis of cell lysates (A and B). Untransduced cells, cells transduced with nonspecific scrambled shRNA, AMPKα-KD cells, or Drp-1-KD cells were incubated in the absence or presence of VacA (250 nM) using pulse toxin exposure. Cells were stained with TMRE for evaluating mitochondrial transmembrane potential (ΔΨ_m_) (C). The data from intoxicated cells were compared relative to unintoxicated cells (measured at 0 h). Statistical significance was determined by comparing data from transduced cells and untransduced cells at the indicated time points. The data were combined from three independent experiments (± SD). Statistical significance (α = 0.05) was determined by comparing transduced cells to untransduced cells and was calculated using two-way ANOVA with Tukey’s multiple comparisons test. *P* < 0.05 indicates statistical significance.

**Fig S6. VacA cellular and subcellular localization.**

AZ-521 cells were collected, and mitochondrial fractions were isolated according to manufacturer’s specifications. Immunoblot analysis was performed to verify the presence of inner mitochondrial membrane and the absence of other cellular markers (lysosome, ER, cytosol, Golgi apparatus) (A). Cells were incubated in the presence of VacA (250 nM) for pulse (B and C) or continuous (D) toxin exposures. VacA in whole cell lysate (W), non-mitochondrial (NM), or mitochondrial (M) fractions was evaluated by immunoblot analysis. Samples in (A) and (B) were normalized by volume. To evaluate non-mitochondrial-associated VacA (C and D), NM fractions were assessed by immunoblot analysis using antibodies specific for VacA and GAPDH (loading control). Quantification of the band intensities was evaluated by densitometry, and the values were compared relative to the 1 h timepoint (C and D). Immunoblots representative of those collected from three independent experiments are shown. The data were combined from three independent experiments (± SD). Statistical significance (α = 0.05) was determined by comparing each timepoint to 1 h using one-way ANOVA with Dunnett’s multiple comparison’s test (C), or AMPKα-KD cells and cells transduced with nonspecific (scrambled) shRNA cells using two-way ANOVA with Sidak’s multiple comparison’s test (D). *P* < 0.05 indicates statistical significance.

**Fig S7: Immunoblot analysis of time-dependent mitochondrial-associated VacA levels.**

Untransduced cells, cells transduced with nonspecific shRNA (scrambled), or cells transduced with AMPKα (AMPKα-KD), or Drp-1 specific shRNA (Drp-1-KD) were incubated in the presence of VacA (250 nM). After continuous toxin exposure, mitochondrial isolations were performed according to the manufacturer’s specifications. Mitochondrial fractions were assessed by immunoblot analysis using antibodies specific for VacA and TIM23 (loading control) to determine the relative levels of mitochondrial-associated VacA. Immunoblots represent those collected from three independent experiments. Quantification of the band intensities was evaluated by densitometry (as shown in Fig. 3D and Fig. 5D).

**Fig S8: VacA-mediated mitochondrial fragmentation is blocked in the presence of Compound C.**

AZ-521 cells were pretreated in the absence (0.1% DMSO) or presence of Compound C (10 µM). After 30 min, cells were further incubated in the absence or presence of Compound C and absence or presence of VacA (250 nM). After 1 h, cells were fixed after 1 h and evaluated by fluorescence microscopy analysis using antibodies specific for TOM20. Fluorescence images (White = TOM20; Blue = DAPI) shown are representative of three independent experiments. Quantification of mitochondrial fragmentation was assessed by determining the percent of cells displaying predominately fragmented mitochondria. Data were combined from three independent experiments (± SD) with at least five fields of view from each condition. Scale bars indicate 10 µm. Statistical significance (α = 0.05) was calculated using an unpaired *t*-test. *P* < 0.05 indicates statistical significance.

**Fig S9: Cellular VacA levels are protected from lysosomal degradation in the presence of Bafilomycin A1.**

AZ-521 cells were incubated in the presence or absence of 10 µM Bafilomycin A1. After 30 min, cells were incubated in the presence of VacA (250 nM) for pulse toxin exposure in the presence or absence of Bafilomycin A1. For evaluating relative cellular VacA levels, whole cell lysates were collected after 2 or 24 h of incubation and evaluated using immunoblot analysis to determine the relative levels of VacA or β-actin (loading control). Quantification of the band intensity was evaluated by densitometry, and the values were compared relative to untreated cells at the 2 h timepoint. VacA was not detected in mitochondrial fractions from cells incubated in the absence of VacA. The data were combined from three independent experiments (± SD). Statistical significance (α = 0.05) was calculated by two-way ANOVA with Tukey’s multiple comparisons test. *P* < 0.05 indicates statistical significance.

**Fig S10: Comparison of VacA-mediated cell death in AZ-521 and AGS cells.**

AZ-521 or AGS cells were incubated in the absence or presence of VacA (10, 35, 250 nM). After 48 h, cells were collected and stained with annexin V, for analysis using flow cytometry according to the manufacturer’s specifications. The data were combined from three independent experiments (± SD). Statistical significance (α = 0.05) was calculated using two-way ANOVA with Sidak’s multiple comparisons test. *P* < 0.05 indicates statistical significance.
